# circKCNN2 suppresses the recurrence of hepatocellular carcinoma at least partially via regulating miR‐520c‐3p/methyl‐DNA‐binding domain protein 2 axis

**DOI:** 10.1002/ctm2.662

**Published:** 2022-01-20

**Authors:** Donghong Liu, Wenbin Liu, Xi Chen, Jianhua Yin, Longteng Ma, Mei Liu, Xinyu Zhou, Linfeng Xian, Peng Li, Xiaojie Tan, Jun Zhao, Yong Liao, Guangwen Cao

**Affiliations:** ^1^ Key Laboratory of Molecular Biology for Infectious Diseases Ministry of Education Chongqing Medical University Chongqing China; ^2^ Institute for Viral Hepatitis Chongqing Medical University Chongqing China; ^3^ Department of Infectious Diseases the Second Affiliated Hospital Chongqing Medical University Chongqing China; ^4^ Department of Epidemiology Second Military Medical University Shanghai China; ^5^ Department of Hepatic Surgery Eastern Hepatobiliary Surgery Hospital Second Military Medical University Shanghai China

**Keywords:** circKCNN2, HCC, lenvatinib, miR‐520c‐3p, recurrence

## Abstract

**Background:**

Recurrence is the major cause of hepatocellular carcinoma (HCC) death. We aimed to identify circular RNA (circRNA) with predictive and therapeutic value for recurrent HCC.

**Methods:**

Tissue samples from recurrent and non‐recurrent HCC patients were subjected to circRNA sequencing and transcriptome sequencing. circKCNN2 was identified through multi‐omics analyses. The effects of circKCNN2 on HCC were evaluated in cells, animals, database of The Cancer Genome Atlas, and a cohort with 130 HCC patients. circRNA precipitation, chromatin immunoprecipitation assay, RNA pull‐down, luciferase assay, and cell experiments were applied to evaluate the interaction of circKCNN2 with miRNAs and proteins. The association between circKCNN2 and the therapeutic effect of lenvatinib was investigated in HCC cell lines and HCC tissue‐derived organoids.

**Results:**

The expression of circKCNN2 was downregulated in HCC tissues and predicted a favorable overall survival and recurrence‐free survival. The expression of circKCNN2 was positively correlated with the parental gene, potassium calcium‐activated channel subfamily N member (KCNN2). Nuclear transcription factor Y subunit alpha (NFYA) was proven to inhibit the promoter activity of KCNN2, downregulate the expression of KCNN2 and circKCNN2, and predict an unfavorable recurrence‐free survival. Ectopic expression of circKCNN2 inhibited HCC cell proliferation, colony formation, migration, and tumor formation in a mouse model. miR‐520c‐3p sponged by circKCNN2 could reverse the inhibitory effect of circKCNN2 on HCC cells and down‐regulate the expression of methyl‐DNA‐binding domain protein 2 (MBD2). The intratumoral expression of MBD2 predicted a favorable recurrence‐free survival. circKCNN2 down‐regulated the expression of fibroblast growth factor receptor 4 (FGFR4), which can be reversed by miR‐520c‐3p and knockdown of MBD2. Lenvatinib inhibited the expression of FGFR4 and upregulated the expression of circKCNN2 and MBD2. Ectopic expression of circKCNN2 in HCC cells enhanced the therapeutic effect of lenvatinib. However, the high inherent level of circKCNN2 in HCC cells was associated with lenvatinib resistance.

**Conclusions:**

circKCNN2, transcriptionally repressed by NFYA, suppresses HCC recurrence via the miR‐520c‐3p/MBD2 axis. Inherent level of circKCNN2 in HCC cells predisposes anti‐tumor effect of lenvatinib possibly because both circKCNN2 and lenvatinib repress the expression of FGFR4. circKCNN2 may be a promising predictive biomarker and therapeutic agent for HCC recurrence.

## Graphical abstract

1. Increased level of circKCNN2 in tumors predicts a favorable overall survival and recurrence‐free survival.

2. circKCNN2 downregulates FGFR4 and inhibits HCC development by regulating miR‐520c‐3p/MBD2 axis.

3. The high inherent level of circKCNN2 may be associated with lenvatinib resistance.

4. NFYA inhibits the expression of circKCNN2 and is associated with an unfavorable recurrence‐free survival.

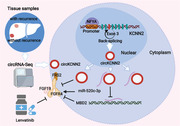

**Abbreviations**
AFP: α‐fetoprotein
AHR: aryl hydrocarbon receptor
CCK8: cell counting kit‐8
ceRNAs: competing endogenous RNAs
circRIP: circular RNA immunoprecipitation
circRNA: circular RNA
FBS: fetal bovine serum
FC: fold change
FCM: flow cytometry
FDR: false discovery rate
FGF19: fibroblast growth factor 19
FGFR4: fibroblast growth factor receptor 4
FRS2: fibroblast growth factor receptor substrate 2
GAPDH: glyceraldehyde‐3‐phosphate dehydrogenase
GEO: gene expression omnibus
GLIS3: GLIS family zinc finger 3
GPC3: glypican 3
GSEA: gene set enrichment analysis
H&E: hematoxylin‐eosin staining
HBV: hepatitis B virus
HCC: hepatocellular carcinoma
IHC: immunohistochemistry
IP: immunoprecipitation
JUNB: Jun B proto‐oncogene
KCNN2: potassium calcium‐activated channel subfamily N member 2
LIHC: liver hepatocellular carcinoma
MBD2: methyl‐CpG binding domain protein 2
miRNA: microRNA
MVI: microscopic vascular invasion
NFYA: nuclear transcription factor Y subunit alpha
OS: overall survival
PMSF: phenylmethanesulfonyl fluoride
RCOR1: RE1 silencing transcription factor corepressor 1
RFS: recurrence‐free survival
RT‐qPCR: quantitative real‐time reverse‐transcription polymerase chain reaction
siRNAs: small interfering RNAs
TCGA: the Cancer Genome Atlas
TFBSs: transcription factor binding sites
TSS: transcription start site
VEGFR: vascular endothelial growth factor receptor

## 1 INTRODUCTION

In 2020, approximately 0.83 million people died from primary liver cancer globally, of which 75%‐85% were hepatocellular carcinoma (HCC).^1^ Chronic infection with hepatitis B virus (HBV) remains a major cause of HCC.^1^, ^2^ Surgical resection remains to be the mainstay of curative treatment. The postoperative recurrence rate of HCC is up to 70%.^3^ Tumor size, satellite nodules, microscopic vascular invasion (MVI), advanced stage, HBV load, inflammation, and increased α‐fetoprotein (AFP) are proven to be important prognostic factors in HCC.^3^, ^4^ Antiviral treatment decreases postoperative recurrence of HBV‐related HCC (HBV‐HCC).^5^, ^6^ However, it remains a great challenge to increase postoperative survival in HCC. Sorafenib and lenvatinib are the first‐line therapies for advanced HCC, both of which inhibit endothelial growth factor receptor (VEGFR) and fibroblast growth factor receptor (FGFR).^7^, ^8^ Compared to sorafenib, lenbatinib shows a stronger capability of improving progression‐free survival.^8^ However, the response rate of lenvatinib in Child‐Pugh A and B HCC patients is only 42.9% and 25.0%, respectively.^9^ All the current clinicopathological markers are not sufficiently accurate and should be complemented with molecular biomarkers.^10^ The molecular biomarkers to guide lenvatinib treatment are also limited.^11^ Exploring novel predictive biomarkers and therapeutic targets will provide specific prophylactic and therapeutic options for postoperative recurrence of HCC.

Circular RNAs (circRNAs) have a circular configuration through a typical 5′ to 3′‐phosphodiester bond, which are more stable than their parental linear mRNAs.^12^ circRNAs regulate biological processes by sponging microRNA (miRNA), regulating transcription, affecting epigenetic modification, and acting as competing endogenous RNAs (ceRNAs).^13^ circRNA also represents a novel direction of harvesting prognostic and predictive biomarkers and therapeutic targets in HCC.^14^ For instances, circSLC3A2 functions as an oncogenic factor in HCC via sponging miR‐490‐3p.^15^ circMET promotes HCC development via inducing an epithelial‐to‐mesenchymal transition.^16^ However, the role of circRNA in the recurrence and drug resistance of HCC remains largely unknown.

Here, we identified a novel circRNA, circKCNN2, that inhibits the recurrence of HCC at least partially via regulating miR‐520c‐3p / methyl‐DNA‐binding domain protein 2 (MBD2) axis. The high inherent level of circKCNN2 may be associated with resistance to lenvatinib. This study indicates that circKCNN2 is a potential therapeutic molecule to treat recurrent HCC.

## 2 MATERIAL AND METHODS

### 2.1 Patients and samples

In total, 143 HBV‐HCC patients who received radical surgery at the Eastern Hepatobiliary Surgery Hospital (Shanghai, China) between February 2011 and September 2012 were recruited in this study. All patients were seropositive for HBV DNA and hepatitis B surface antigen and pathologically diagnosed as HCC. The enrolled patients did not receive any other anticancer treatments before and after the operation. The follow‐up examination was carried out as previously described.^5^ The last follow‐up date was August 23, 2018. Of all 143 HCC patients, thirteen were selected as a training cohort for deep‐sequencing analysis. Of the 13 patients, nine did not relapse within 5 years after the surgery, while four relapsed after the first surgery. The tumor tissues and corresponding adjacent tissues of all 13 patients were subjected to deep‐sequencing analysis. The tissue samples of the four relapsing patients were collected from the first surgery. The remaining 130 HCC patients served as a validation cohort. In both of training cohort and validation cohort, no significant difference was evident in age, gender, tumor stage, and fibrosis levels between patients with recurrence and those without recurrence (Table S1 and Table S2). For the construction of organoids, two HCC tissues were collected during surgery in September 2018 and February 2019, respectively. Corresponding clinical data and follow‐up information were also collected. All participants were self‐reported Han Chinese and provided written informed consents. The study protocol conformed to the ethical guidelines of the 2000 Declaration of Helsinki and was approved by the ethics committees of the Second Military Medical University and the Eastern Hepatobiliary Surgery Hospital.

### 2.2 circRNA sequencing (circRNA‐seq) and mRNA sequencing (mRNA‐seq)

Total RNA was extracted from tumors and adjacent tissues. The sequencing libraries for the mRNA‐seq and circRNA‐seq were constructed using TruSeq Stranded Total RNA with Ribo‐Zero Gold Kit (Illumina, San Diego, CA) and Ribonuclease R Kit (Lucigen, Middleton, WI). The HiSeqTM 2500 sequencing platform (Illumina) was applied for circRNA‐seq and mRNA‐seq. Details for the identification of circRNA, differential expression analysis, and gene sets enrichment analysis (GSEA) are available in the Supplementary material. The data of circRNA‐seq and mRNA‐seq are available in the Gene Expression Omnibus (GEO) database under the accession number of GSE129687 and GSE129689, respectively.

### 2.3 Transcription factor binding sites (TFBSs) analysis

The MotEvo‐predicted TFBSs within the promoter of potassium calcium‐activated channel subfamily N member (KCNN2) were downloaded from the database of SwissRegulon (https://swissregulon.unibas.ch/sr/).^17^ The hg19 genomic coordinates were converted to the hg38 ones by the liftover tool (https://genome.ucsc.edu/cgi‐bin/hgLiftOver). TFBSs whose posterior probabilities > 0.8 were kept for further analysis. The expression profiles of these transcription factors were retrieved from The Cancer Genome Atlas (TCGA) database (https://xenabrowser.net/) to validate the expression changes identified in our data.

### 2.4 Cell culture

Human HCC cell lines (Huh7 and HepG2) and HEK293T cell line were purchased from the Chinese Academy of Sciences (Shanghai, China). Before the experiments, all cell lines were authenticated using the genotyping analysis of short tandem repeat by Biowing Biotechnology (Shanghai, China). All cell cultures were tested for mycoplasma contamination every 3 months. Small interfering RNA (siRNAs) against circKCNN2, JunB proto‐oncogene (JUNB), nuclear transcription factor Y subunit alpha (NFYA), and MBD2 were synthesized by GenePharma (Shanghai, China). The mimics and inhibitors of miR‐520c‐3p were purchased from GenePharma. The sequences of siRNAs, miRNA mimics, and miRNA inhibitors were listed in Table S3 and Table S4. The overexpression lentivirus of circKCNN2 and the negative control lentivirus were purchased from Obio Technology (Shanghai, China). Details for cell culture, transfection, construction of recombinant lentiviruses, and lentivirus infection are available in the Supplementary material.

### 2.5 Cell proliferation, colony formation, and migration assay

Cell proliferation was assessed by the Cell Counting Kit‐8 (CCK8) kit (Dojindo, Osaka, Japan) according to the instruction manuals. For colony formation assay, 500 cells were seeded into 6‐well plates and were incubated for 21 days. Cell clones were stained with crystal violet and counted manually. The experiments were performed in triplicate. The ability of cell migration was measured with Transwell inserts (Corning, New York, NY). Details for CCK8 assay and transwell assay are available in the Supplementary material.

### 2.6 Cell cycle assay

The cell cycle assay was performed with flow cytometry (Merck Millipore, Rockville, MD). Cells were digested and fixed at ‐20°C in 70% ethanol overnight. The cell cycle distribution was measured by flow cytometry using the Propidium Iodide (PI)/RNase staining buffer (BD, San Diego, CA) and was analyzed by ModFit LT software (Verity Software House, Topsham, ME). Each assay was performed in triplicate.

### 2.7 Prediction of circRNA targets and miRNA targets

The potential targets of circKCNN2 were predicted by the RegRNA2.0 database (http://regrna2.mbc.nctu.edu.tw/). Potential targets of valid miRNAs were predicted by miRDIP (https://www.biostars.org/p/317966/) and miRTarbase (https://maayanlab.cloud/Harmonizome/resource/MiRTarBase).

### 2.8 Luciferase reporter assay

To determine the transcription factors regulating the expression of KCNN2, the promoter region (1000 bp upstream of the transcription start site) of KCNN2 was synthesized and cloned into pGL4.10 plasmid by Obio Technology. To confirm the binding between circKCNN2 and miRNAs, the linear sequences of wild‐type and mutant circKCNN2 were synthesized and cloned into the pMIR‐REPORT vector. These constructed reporters were named circKCNN2‐WT and circKCNN2‐Mut, respectively. The luciferase reporter assay was also applied to evaluate the binding of miRNA to the 3′UTR regions of MBD2 and GLIS family zinc finger 3 (GLIS3), respectively. The wild‐type and mutant 3′UTR sequences of MBD2 and GLIS3 were synthesized and cloned into the pMIR‐REPORT luciferase vector. In the mutant 3′UTR of MBD2 and GLIS3, the sequences of all putative miR‐520c‐3p binding sites were replaced by scramble sequences. These constructed reporters were named MBD2‐WT, MBD2‐Mut, GLIS3‐WT, and GLIS3‐Mut, respectively. The constructed reporter, renilla control reporter, and the miRNAs targeting transcription factors or mimics of miR‐520c‐3p were co‐transfected into cells. Dual‐luciferase reporter assay was carried out 24 h later as previously described.^18^ The sequences of constructed plasmids and mutation sites were listed in Table S5.

### 2.9 Chromatin immunoprecipitation (ChIP) assay

ChIP assay was performed by using ChIP‐IT Express Enzymatic Kit (Active Motif, Carlsbad, CA) according to the manufacturer's instruction. For the ChIP assay of NFYA, HepG2 cells were transfected with the overexpression plasmid of GFP tagged NFYA (GFP‐NFYA, Sino Biological, Beijing, China) or the corresponding vector plasmid (GFP‐vector) 24h before ChIP assay. GFP‐Trap Agarose (Chromotec GmbH, Karlsruhe, Germany) was applied for immunoprecipitation. HepG2 cells were directly subjected to the ChIP assay of JUNB and chromatin fragments were immunoprecipitated with the antibody against JUNB (#3753, CST, Danvers, MA). A normal rabbit IgG (#2729, CST) was used as the non‐specific antibody control of immunoprecipitation. The total DNA (input) and ChIP‐enriched DNA were subjected to quantitative PCR (qPCR). The enrichment value was represented as a percentage of input. Primers were listed in Table S6.

### 2.10 circRNAs precipitation (circRIP) assay and RNA pull‐down assay

miRNAs binding to circKCNN2 was identified using the circRIP as previously described.^19^ Briefly, HepG2 cells were transfected with biotin‐labeled circKCNN2 probes (biotin‐circKCNN2) (RiboBio, Guangzhou, China) or negative control probes (biotin‐scramble) (RiboBio). Then, cells were fixed with formaldehyde, lysed, sonicated, and centrifugated. For each group, 50μL supernatant was kept as input control. The remaining supernatant was incubated with dynabeads M280 streptavidin (Invitrogen, Dynal, Oslo). The mixture was washed and incubated with proteinase K (Beyotime, Shanghai, China). The RNA level was quantified by Real‐Time Quantitative Reverse Transcription PCR (RT‐qPCR). The group of input and biotin‐scramble severed as a normalization control and negative control, respectively. The enrichment level of miR‐520c‐3p was presented as a percentage of input (circRIP/input ratio). For RNA pull‐down assay, biotin‐labeled miR‐520c‐3p probe (biotin‐miR‐520c‐3p) (RiboBio) or negative control (biotin‐NC) were transfected into HepG2 cells. The remaining procedures were identical to those in circRIP. The enrichment value was represented as a percentage of input.

### 2.11 RT‐qPCR, western blot, and immunohistochemistry

Trizol reagent (Invitrogen) was applied to extract total RNA from tissue samples and HCC cells. The transcript abundance of circKCNN2 and genes were quantified by using PrimeScript RT Master Mix (Takara, Dalian, China) and TB Green Premix Ex Taq II (Takara), respectively. The Bugle‐Loop™ miRNA qPCR kit (RiboBio) was applied to quantify miRNA‐520c‐3p. Glyceraldehyde‐3‐phosphate dehydrogenase (GAPDH) served as internal reference for the quantification of gene and circRNA while U6 was applied as the internal reference for the quantification of miRNA. All the primers used for RT‐qPCR assays are listed in Table S6. Western blot and IHC assays were conducted as previously described.^20^ The primary antibodies used in this study were as follows: MBD2 (1:1000, # A301633A, Bethyl, Montgomery, TX), FGFR4 (1:1000, #8562, CST, Danvers, MA), FGF19 (1:1000, #83348, CST), FRS2 (1:1000, #MAB4069, R&D Systems, Minneapolis, MN), and GAPDH (1:1000, #3683s, CST). Western blot analyses were repeated three times. GAPDH was used as the loading control and normalization control. ImageJ software (version 1.51, https://imagej.nih.gov/ij/) was applied to quantify the signal strength of the bands.

### 2.12 Animal research

Male non‐obese diabetic server combined immune deficiency (Nod‐SCID) mice (5‐week‐old) were purchased from Jihui laboratory animal care cooperation (Shanghai, China). Ten mice were randomly assigned to each group. A total of 1.5 × 10^6^ Huh7 cells expressing circKCNN2 or parental cells infected with lentivirus negative control were mixed with matrigel and injected into mice subcutaneously. Ten days after injection, the siRNAs against circKCNN2 or the siRNA with scrambled sequence were injected into the tumor bodies twice a week at a concentration of 20 nM in 100μL PBS. Tumor growth was measured every other day. Tumor volume was calculated with the formula: V = length × width^2^/2. Five weeks later, tumors were harvested for hematoxylin‐eosin (H&E) staining and IHC. All animal experimental procedures were approved by the Ethics Committee of the Second Military Medical University.

### 2.13 Lenvatinib treatment and cell apoptosis

HepG2 and Huh7 cells were treated with different concentrations of lenvatinib (Selleck, Ibaraki, Japan) for 2 days. Cell viability was detected using CCK8 assay. Cell apoptosis was tested by flow cytometry (FCM) using APC‐Annexin V (Biolegend Inc., San Diego, CA) and 7‐AAD (Biolegend Inc.). The CytExpert Software (version 2.0, Beckman Coulter) was used for data acquisition and analysis. Each cytometry assay was conducted in triplicate.

### 2.14 Generation of human HCC organoids

Human HCC organoids were constructed as previously described.^21^ A total of 2000 organoid cells were seeded in a 96‐well plate with 7 μl Matrigel matrix droplets (Corning Life Sciences, Tewksbury, MA). The organoids were then treated with the medium containing 35 μM lenvatinib (Selleck) for 6 days. Organoids were visualized using a bright field microscope at 10× magnification. Five randomly chosen visual fields were accessed per well and each experiment group was repeated with 3 wells. The diameters of organoids were quantified using ImageJ software.

### 2.15 Statistical analysis

Survival analysis was conducted by using Kaplan‐Meier test and log‐rank test. The R package maxstat was adopted to find the optimal cut‐off value for the expression level of circKCNN2 in HCC. The difference in continuous variables was determined by Student's *t*‐test. For non‐normal data, the Wilcoxon sum rank test was used. The TFBSs prediction and the analyses of circRNome and transcriptome data were performed with R platform version 4.0.2 (Core Development Team, http://www.r‐project.org/). The survival analyses of the validation cohort data were performed with SPSS version 23.0 (SPSS, Chicago, IL). The cut‐off value was determined using X‐tile software (http://medicine.yale.edu/lab/rimm/research/software.aspx). The analyses for the data of in vitro and in vivo experiments were performed with GraphPad Prism version 5.0 (GraphPad Software, San Diego, CA). *P *< 0.05 was considered as statistically significant.

## 3 RESULTS

### 3.1 Identification of functional circRNAs related to HCC recurrence

**FIGURE 1:**
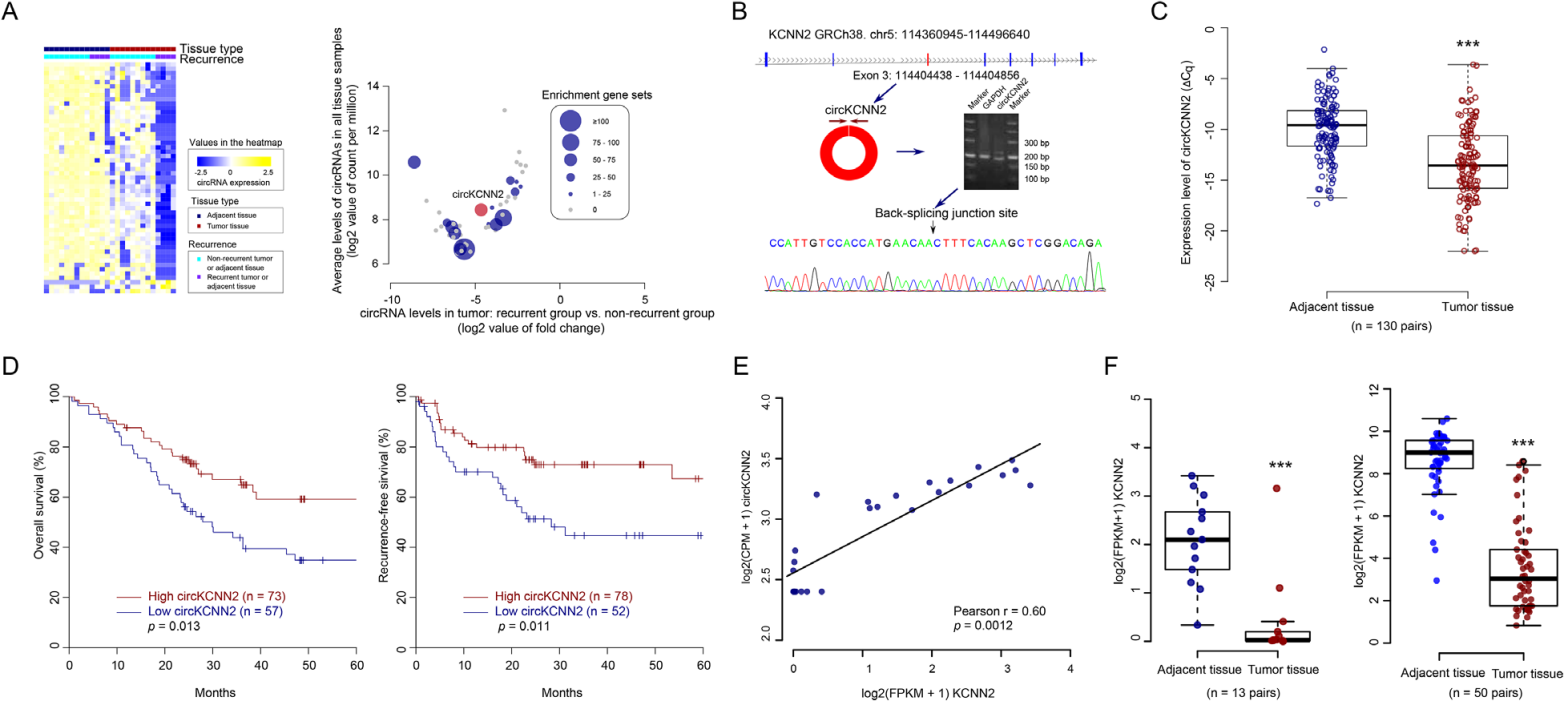
The identification of circKCNN2. (A) Left: heat map of differently expressed circRNA. Right: the plot of gene set enrichment analysis for differently expressed circRNAs. The expression level of circRNAs in tumors, fold changes of circRNAs between tumors from two groups of HCC patients, and the number of enriched gene sets was plotted together. (B) The structure diagram of *KCNN2* and circKCNN2. The coding region of circKCNN2 is overlapped with exon 3 of *KCNN2*. The back‐splicing junction site of circKCNN2 was validated through PCR and Sanger sequencing. (C) The expression level of circKCNN2 in the tumor and adjacent tissues of 130 HCC patients. (D) Kaplan‐Meier curves of overall survival and recurrence‐free survival for the patients with different levels of circKCNN2. (E) The expression of linear *KCNN2* was significantly correlated with the expression of circKCNN2. (F) KCNN2 was down‐regulated in the tumors compared to the adjacent tissues. Left: the mRNA‐seq data of the discovery cohort. Right: the mRNA‐seq data of TCGA liver hepatocellular carcinoma (LIHC) cohort. *P < 0.05; **P < 0.01; ***P < 0.001

Firstly, differential expression analysis was performed using the circRNome data of 13 HBV‐related HCC patients. We obtained 53 circRNA candidates that were differentially expressed between tumors and adjacent tissues as well as between tumors from relapsing patients and tumors from non‐relapsed patients (Figure 1A, left). Next, the correlation between circRNA expression and gene expression was calculated to identify the genes significantly related to each circRNA candidate. The circRNA‐related genes were subjected to GSEA. Fifteen potentially functional circRNAs that had at least one significantly enriched gene set were selected from the 53 circRNA candidates (Figure 1A, right). Among the 15 circRNAs, circKCNN2 (also known as hsa_circ_0127664) was previously reported to be significantly downregulated in brain tissue of patients with progressive glioma, suggesting it may be a tumor suppressor.^22^ Therefore, circKCNN2 was selected for further investigation as a potential suppressor for HCC recurrence. The expression level of circKCNN2 was significantly lower in HCC tissues than in adjacent tissues [fold change (FC) = ‐11.27, false discovery rate (FDR) < 0.001]. The expression of circKCNN2 was also significantly down‐regulated in the primary HCC tissues from relapsing patients, compared to the HCC tissues from non‐relapsed patients (FC = ‐25.15, FDR = 0.044). By GSEA, circKCNN2 significantly enriched 63 gene sets (Table S7). The sequencing data indicated that circKCNN2 might be transcribed from the third exon of KCNN2 (transcript number NM_021614) and then back‐spliced into a circular structure. Through PCR and subsequent Sanger sequencing, the back‐splicing junction site was successfully validated (Figure 1B). The effect of circKCNN2 on the progression of HCC was further estimated in the validation cohort of 130 HBV‐HCC patients enrolled in this study. The expression of circKCNN2 was significantly downregulated in the tumors, compared to that in the adjacent liver tissues (*P *< 0.001) (Figure 1C). Furthermore, elevated circKCNN2 expression level in tumors significantly predicted favorable overall survival (OS) (*P *= 0.013) and recurrence‐free survival (RFS) (*P *= 0.011) (Figure 1D). These data support that the high level of circKCNN2 is significantly associated with a decreased risk of HCC recurrence.

### 3.2 Identification of transcriptional factors regulating the expression of circKCNN2

**FIGURE 2:**
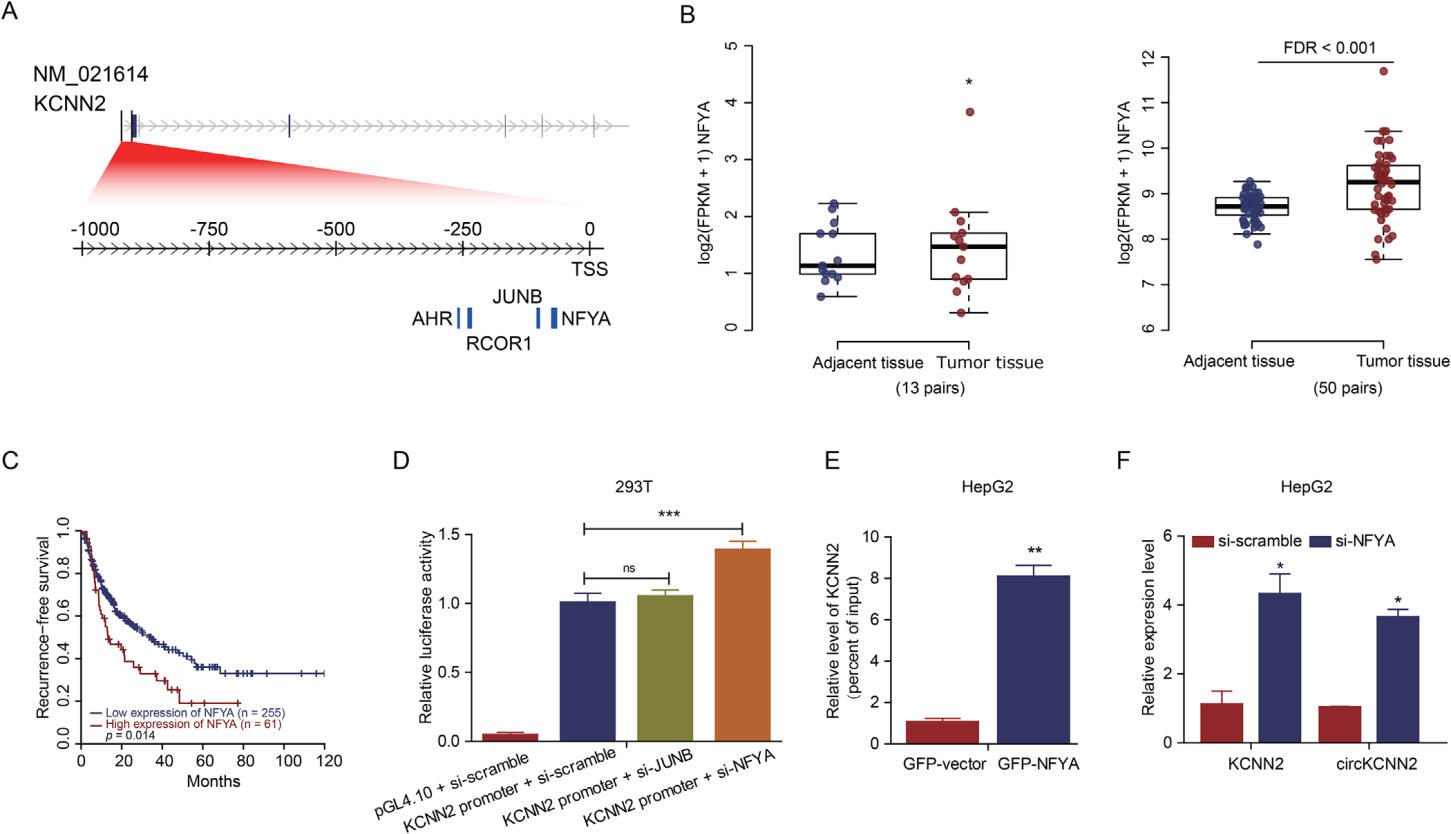
The identification of the functional transcription factor regulating KCNN2 and circKCNN2. (A) The putative transcription factors (posterior probabilities > 0.8) and their binding sites. NFYA: ‐64 bp to ‐76 bp; JUNB: ‐98 bp to ‐105 bp; RCOR1: ‐232 bp to 241 bp; AHR: ‐256 bp to ‐261 bp. (B) The expression levels of NFYA in the tumor and adjacent tissues. Left: in the training cohort of this study. Right: in the TCGA LIHC cohort. (C) The association between the intratumor level of NFYA and the recurrence‐free survival of HCC patients from LIHC cohort. (D) Results of luciferase assays. pGL4.10, empty vector of luciferase reporter; KCNN2 promoter, the luciferase reporter containing the sequence of KCNN2 promoter; si‐scramble/si‐JUNB/si‐NFYA, siRNA targeting scramble sequence/JUNB/NFYA. The group of KCNN2 promoter + si‐scramble served as a reference to calculate a relative value of luciferase activity. (E) Results of ChIP followed qPCR testing the enrichment level of KCNN2 promoter in the DNA pulled down by GFP‐Trap Agarose. GFP‐NFYA/‐vector, DNA from cells transfected with the overexpression plasmid of GFP tagged NFYA/the control vector. The group of GFP‐vector served as a reference to calculate a relative enrichment. (F) RT‐qPCR results displaying the levels of *KCNN2* and circKCNN2. The groups of si‐scramble served as references to calculate relative RNA levels. Cellular experiments were performed in triplicate. N = 3 for each group. ns, no statistically significant; *P < 0.05; **P < 0.01; ***P < 0.001

Based on the mRNA‐seq and circRNA‐seq data, a significant positive association was identified between the expression of circKCNN2 and its parental gene, linear KCNN2 (Pearson coefficient = 0.61, *P* < 0.001) (Figure 1E). The expression of linear KCNN2 was lower in the tumors than in adjacent liver tissues in our data. The same was true in TCGA data (Figure 1F). To elucidate the transcriptional regulation of KCNN2, we downloaded potential TFBSs within 1000 bp upstream of KCNN2's transcription start site (TSS). We investigated the TFBSs with posterior probability of > 0.8. Four transcriptional factors were predicted to bind 4 loci within 1000 bp of the KCNN2 promoter region (Figure 2A). Of those transcriptional factors, NFYA and JUNB showed significant expression changes and significant effects on recurrence. NFYA was significantly up‐regulated in the tumors compared with that in adjacent liver tissues both in our data and in the TCGA liver hepatocellular carcinoma (LIHC) database (Figure 2B). The high level of NFYA in the tumor was significantly associated with an unfavorable RFS (Figure 2C). JUNB was significantly down‐regulated in the tumors compared to that in adjacent liver tissues both in our data and in the TCGA LIHC database (Figure S1A, S1B). Besides, the high level of JUNB in the tumor was associated with a favorable RFS (Figure S1C).

We further confirm the functions of these two putative transcriptional factors. The luciferase assay showed that the promoter activity of KCNN2 was significantly enhanced by the knockdown of NFYA rather than JUNB (Figure 2D). ChIP‐qPCR results demonstrated that the KCNN2 promoter region directly bonded to NFYA rather than JUNB (Figure 2E and Figure S1D). Knocking down NFYA significantly upregulated the expression of KCNN2 and circKCNN2 (Figure 2F), whereas knockdown of JUNB had no obvious effect on the levels of KCNN2 and circKCNN2 (Figure S1E). Thus, NFYA should be the key transcriptional repressor for linear KCNN2 and circKCNN2.

### 3.3 The effects of circKCNN2 on the progression of HCC in vitro and in vivo

**FIGURE 3:**
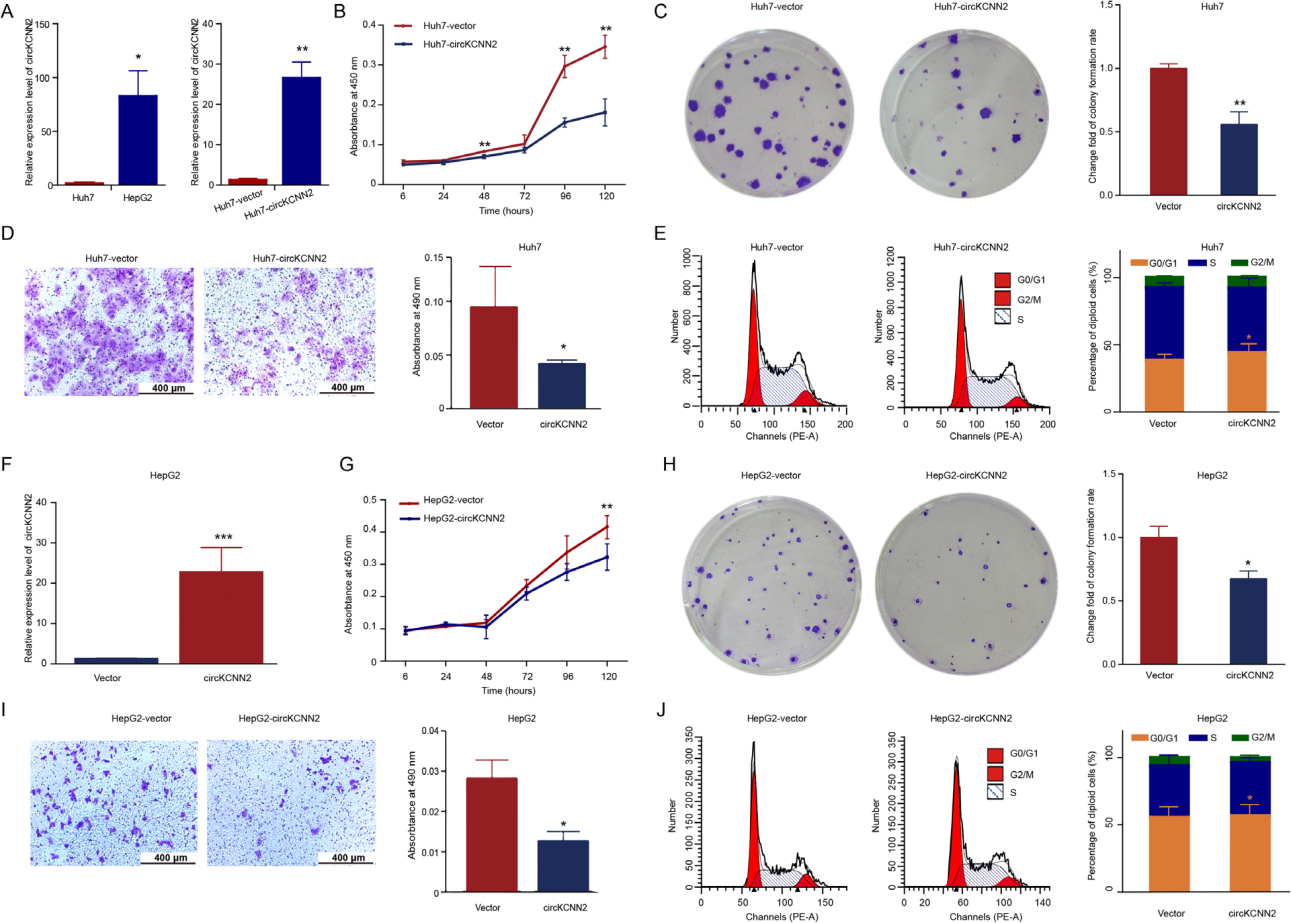
The effect of ectopic overexpression of circKCNN2 on the proliferation, colony formation, and migration of Huh7 and HepG2 cells. (A) RT‐qPCR results of circKCNN2 expression. Left, the inherent level of circKCNN2 in Huh7 and HepG2 cells. Huh7 cells served as a reference to calculate relative expression levels. Right, ectopic overexpression of circKCNN2 in Huh7 cells. Huh7‐vector (reference for normalization), cells infected with control lentivirus; Huh7‐circKCNN2, cells infected with circKCNN2 overexpression lentivirus. (B) CCK8 assays showed that ectopic overexpression of circKCNN2 inhibited the proliferation of Huh7 cells. (C) The colony formation was inhibited in Huh7‐circKCNN2 cells. (D) Transwell assays showed that ectopic overexpression of circKCNN2 inhibited the migration of Huh7 cells. (E) The rate of cells in G_0_/G_1_ was significantly higher in Huh7‐circKCNN2 cells than that in Huh7‐vector cells. (F) The expression level of circKCNN2 in HepG2 cells. HepG2‐vector, cells infected with control lentivirus; HepG2‐circKCNN2, cells infected with circKCNN2 overexpression lentivirus. (G) Ectopic overexpression of circKCNN2 inhibited the proliferation of HepG2 cells. (H) The colony formation was inhibited in HepG2‐circKCNN2 cells. (I) Ectopic overexpression of circKCNN2 inhibited the migration of HepG2 cells. (J) The rate of cells in G_0_/G_1_ was significantly higher in HepG2‐circKCNN2 cells than that in HepG2‐vector cells. All the assays were performed at least three times. N = 3 for each group. *P < 0.05, **P < 0.01, ***P < 0.001

We firstly measured the inherent level of circKCNN2 in Huh7 and HepG2 cell lines using RT‐qPCR. Huh7 showed a significantly lower level of circKCNN2 than did HepG2 (Figure 3A left). Stable overexpression of circKCNN2 was induced by lentiviral infection in Huh7 cells (Figure 3A right). The effects of circKCNN2 on cell proliferation, migration, and cell cycle distribution were initially evaluated in Huh7 cells. Cell growth and colony formation ability were significantly suppressed by circKCNN2 overexpression (Figure 3B, C). Transwell assay showed that cell migration was significantly decreased by circKCNN2 (Figure 3D). Cell cycle assays indicated that circKCNN2 induced cell cycle arrest at the G_0_/G_1_ phase (Figure 3E). The experimental results in Huh7 cells were repeatable in HepG2 (Figure 3F–J). Thus, circKCNN2 inhibits cell proliferation, migration, colony formation, and cell cycle progression in HCC.

**FIGURE 4:**
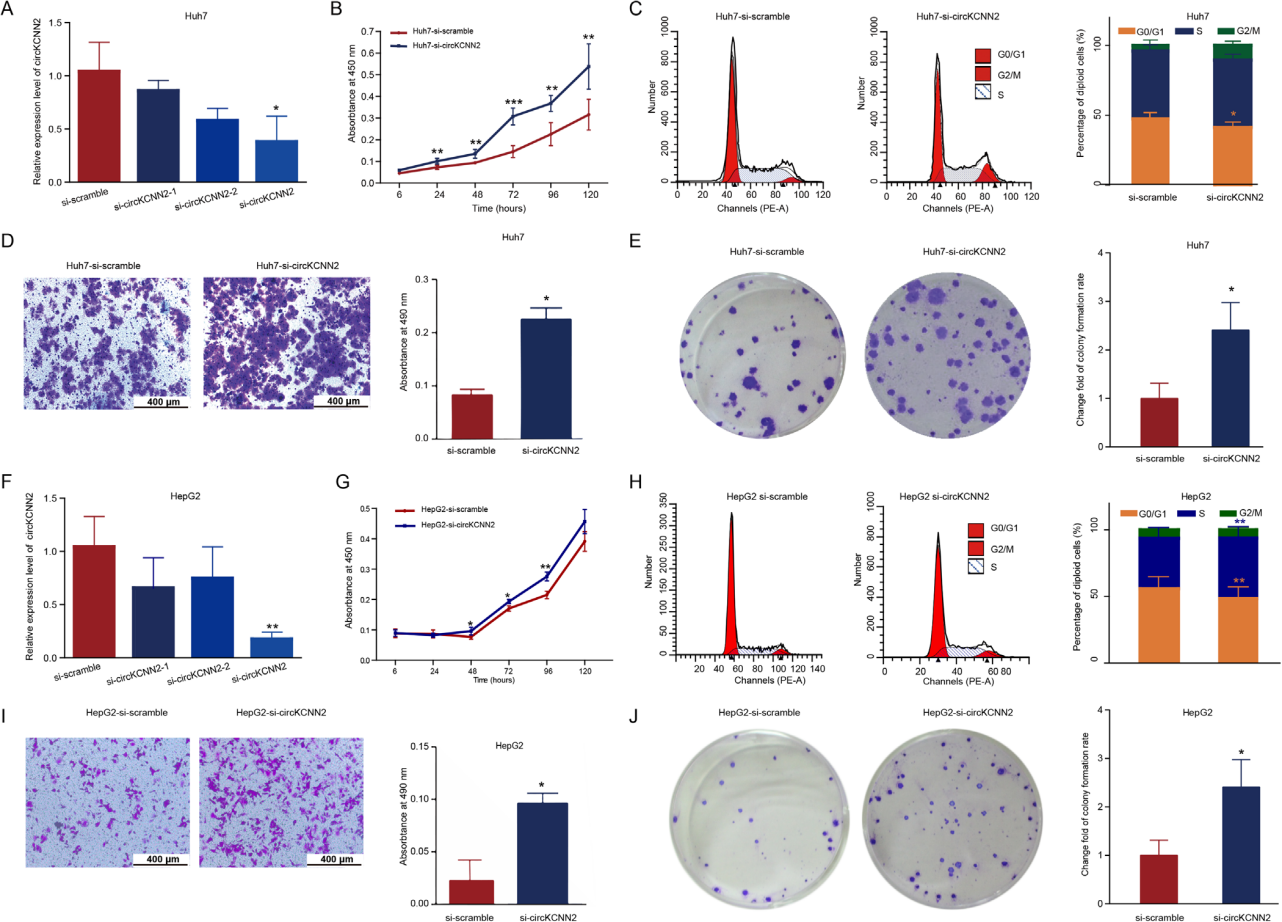
The effect of circKCNN2 knockdown on the proliferation, colony formation, and migration of Huh7 and HepG2 cells. (A) The efficiency of circKCNN2 knockdown in Huh7 cells. si‐circKCNN2: co‐infection with si‐circKCNN2‐1 and si‐circKCNN2‐2. Si‐scramble transfected group served as a reference for normalization. (B) Cell proliferation of the Huh7 cells with circKCNN2 knockdown (Huh7‐si‐circKCNN2) and the control cells (Huh7‐si‐scramble). (C) The proportion of cells in G_0_/G_1_ was significantly lower in Huh7‐si‐circKCNN2 than that in control cells. (D) Knockdown of circKCNN2 promoted migration of Huh7 cells. (E) Knockdown of circCKNN2 promoted the colony formation of Huh7 cells. (F) The efficiency of circKCNN2 knockdown in HepG2 cells. (G) Cell proliferation of the HepG2‐si‐circKCNN2 cells and HepG2‐si‐scramble cells. (H) The proportion of cells in G_0_/G_1_ was significantly lower in HepG2‐si‐circKCNN2 than that in control cells. (I) Knockdown of circKCNN2 promoted migration of HepG2 cells. (J) Knockdown of circCKNN2 promoted the colony formation of HepG2 cells. All the assays were performed at least three times. N = 3 for each group. *P < 0.05; **P < 0.01; ***P < 0.001

To further understand the biological functions of circKCNN2 in HCC, siRNAs targeting the back‐splicing sequence of circKCNN2 were transfected into HCC cells. It was confirmed that co‐transfection with si‐circKCNN2‐1 and si‐circKCNN2‐2 yielded a better knockdown result than did each siRNA alone (Figure 4A). Therefore, in the following experiments, circKCNN2 was knocked down by using both siRNAs. In Huh7 cells, circKCNN2 knockdown significantly upregulated the cell proliferation, increased the proportion of S phase cells, promoted migration, and enhanced the colony formation capacity (Figure 4B–E). The experimental results in Huh7 cells were repeatable in HepG2 (Figure 4F–J).

**FIGURE 5:**
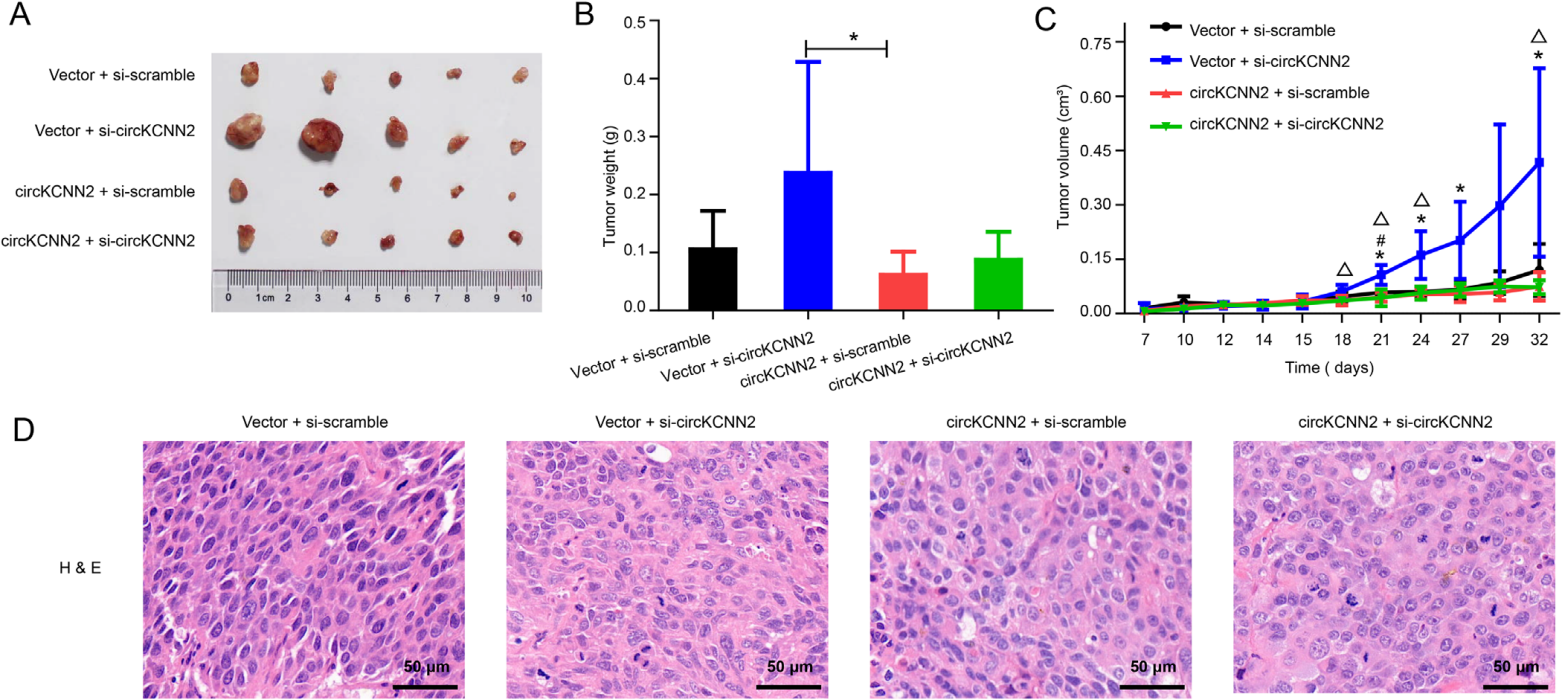
circKCNN2 inhibited tumor growth in vivo. (A) The size of tumors. (B) The weights of tumors among different groups. (C) Growth curve of tumor weighs. (D) Hematoxylin‐eosin (H&E) staining of tumor tissues. Vector, infected with control lentivirus; circKCNN2, infected with circKCNN2 overexpression lentivirus; si‐scramble, injected with siRNA targeting scramble sequencing; si‐circKCNN2, injected with siRNA targeting circKCNN2. *, vector + si‐scramble *vs* vector + si‐circKCNN2, *P <* 0.05; ^#^vector + si‐scramble *vs* circKCNN2 + si‐scramble, *P <* 0.05; △, vector + si‐circKCNN2 *vs* circKCNN2 + si‐circKCNN2, *P <* 0.05

The effects of circKCNN2 overexpression and knockdown were also investigated in vivo. Figure 5 shows the results of tumor transplantation experiments in Nod‐SCID mice. The tumor weight and size were the smallest in the group with circKCNN2 overexpression, biggest in the group with circKCNN2 knockdown. Silencing circKCNN2 in tumors reversed the tumor‐suppressing effects of circKCNN2 (Figure 5A–C). The representative H&E staining was represented in Figure 5D.

### 3.4 miRNAs sponged by circKCNN2 in HCC cells

**FIGURE 6:**
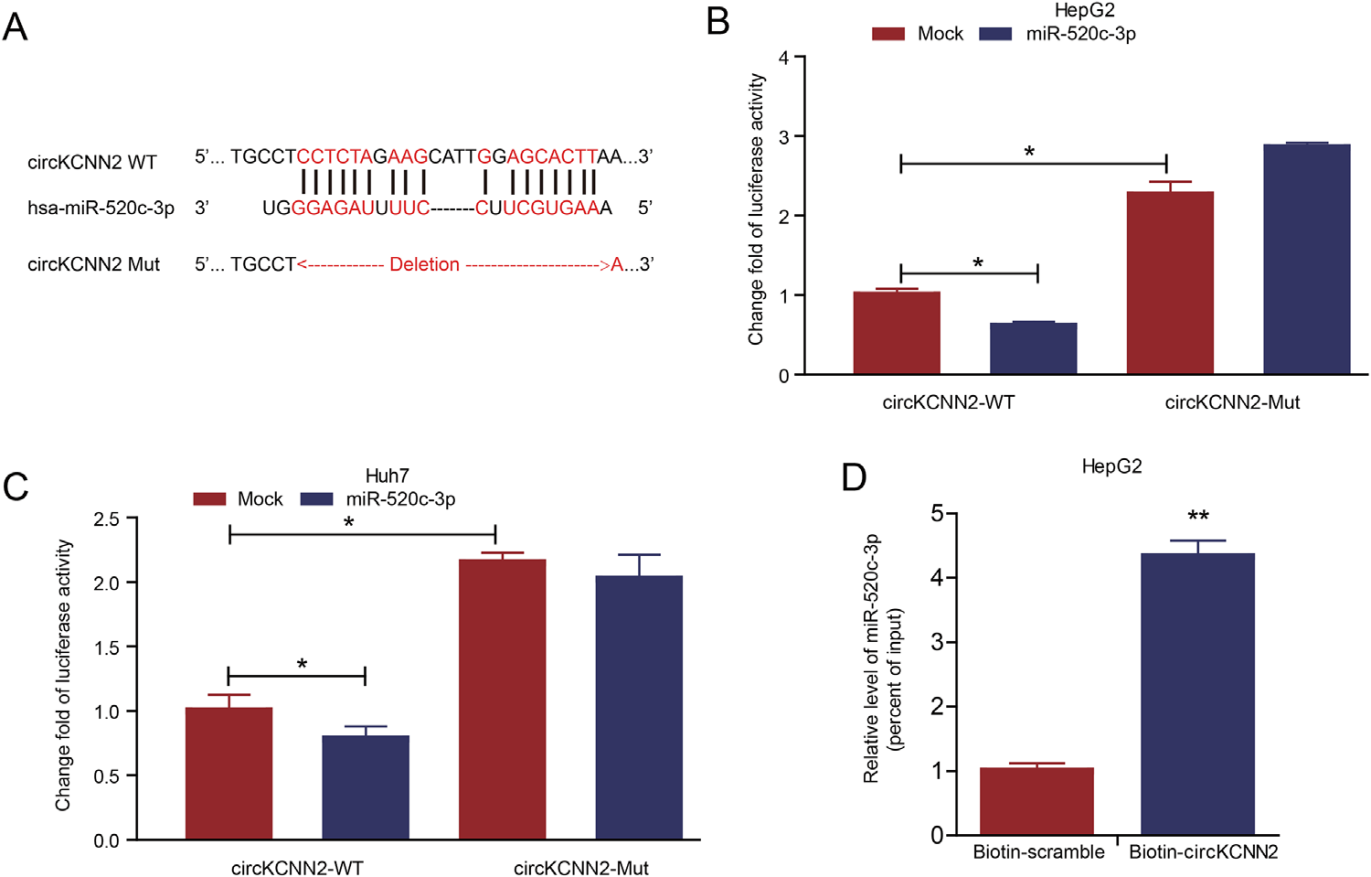
Confirmation of the binding between miR‐520c‐3p and circKCNN2. (A) The predicted binding sites between miR‐520c‐3p and circKCNN2. (B, C) The luciferase activities in HepG2 and Huh7 cells after co‐transfection with the reporter plasmids and miR‐520c‐3p mimics. Mock, miRNA negative control; miR‐520c‐3p, mimics of miR‐520‐3p. The group of circKCNN2‐WT + Mock served as a reference to calculate change fold value. (D) The amount of miR‐520c‐3p in the RNA pulled down by using the biotin‐circKCNN2 probe. The group of biotin‐scramble served as a reference for calculating relative value. All the assays were performed at least three times. N = 3 for each group. *P < 0.05, **P < 0.01, ***P < 0.001

**FIGURE 7:**
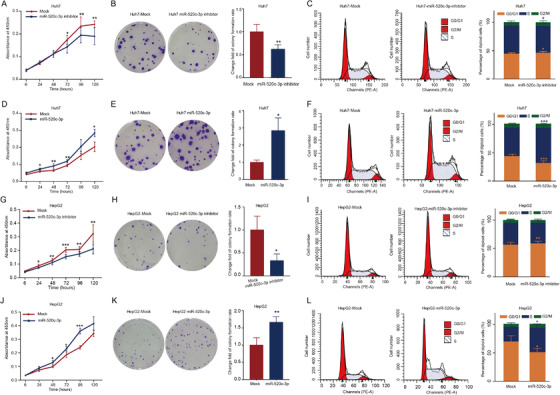
The effects of miR‐520c‐3p on the proliferation, colony formation, and cell cycle of Huh7 and HepG2 cells. (A, B) The transfection of miR‐520c‐3p inhibitor inhibited the proliferation and colony formation of Huh7 cells. (C) Cell cycle distribution in Huh7 cells transfected with miR‐520c‐3p inhibitor. (D, E) The transfection of miR‐520c‐3p miimics promoted the proliferation and colony formation of Huh7 cells. (F) The rate of cells in G_0_/G_1_ was significantly lower in Huh7 cells transfected with miR‐520c‐3p mimics. (G, H) The transfection of miR‐520c‐3p inhibitor inhibited the proliferation and colony formation of HepG2 cells. (I) Cell cycle distribution in HepG2 cells transfected with miR‐520c‐3p inhibitor. (J, K) The transfection of miR‐520c‐3p mimics promoted the proliferation and colony formation of HepG2 cells. (L) The rate of cells in G_0_/G_1_ was significantly lower in HepG2 cells transfected with miR‐520c‐3p mimics. All the assays were performed at least three times. N = 3 for each group. *P < 0.05, **P < 0.01, ***P < 0.001

Targeted miRNAs of circKCNN2 were predicted via querying the RegRNA2.0 (Table S8). Among the miRNA candidates, miR‐520c‐3p was selected due to the highest score of sequence complementarity. In the validation cohort, the level of miR‐520c‐3p was significantly higher in HCC tissues than in adjacent tissues. Compared with the tumor tissue from patients without recurrence, tumors from patients with recurrence showed a significantly higher level of miR‐520c‐3p (Figure S2). Dual‐luciferase reporter assays were performed to confirm whether miR‐520c‐3p was the direct target of circKCNN2. The reporter vectors were constructed as described in the materials and methods section. The relative luciferase activity in cells transfected with circKCNN2‐WT, rather than in cells transfected with circKCNN2‐Mut, was significantly down‐regulated when co‐transfected with miR‐520c‐3p mimics. Furthermore, the luciferase activity was significantly lower in HCC cells transfected with circKCNN2‐WT than in those transfected with circKCNN2‐Mut (Figure 6A–C). Moreover, circRIP assay demonstrated a specific enrichment of miR‐520c‐3p in the RNA pulled down by using the biotin‐circKCNN2 probe, compared with the RNA pulled down by the control probe targeting scramble sequence (Figure 6D). These data indicated that miR‐520c‐3p was the direct target of circKCNN2. To further investigate the functions of miR‐520c‐3p in HCC cells, miR‐520c‐3p inhibitors and miR‐520c‐3p mimics were transfected into HCC cell lines, respectively. The expression of miR‐520c‐3p in Huh7 and HepG2 cells following the transfection was confirmed by RT‐qPCR (Figure S3). miR‐520c‐3p inhibitor significantly suppressed the cell growth and the colony‐forming capacity of Huh7 cells (Figure 7A–B). Cell cycle distribution assays revealed that the proportion of cells at the S phase were significantly decreased by miR‐520c‐3p inhibitor (Figure 7C). Conversely, miR‐520c‐3p mimics had significantly opposite effects (Figure 7D–F). The experimental results in Huh7 cells were repeatable in HepG2 cells (Figure 7G–L). These results indicate that miR‐520c‐3p promotes the proliferation of HCC cells.

### 3.5 Reversal of circKCNN2's effects on HCC cells by miR‐520c‐3p

**FIGURE 8:**
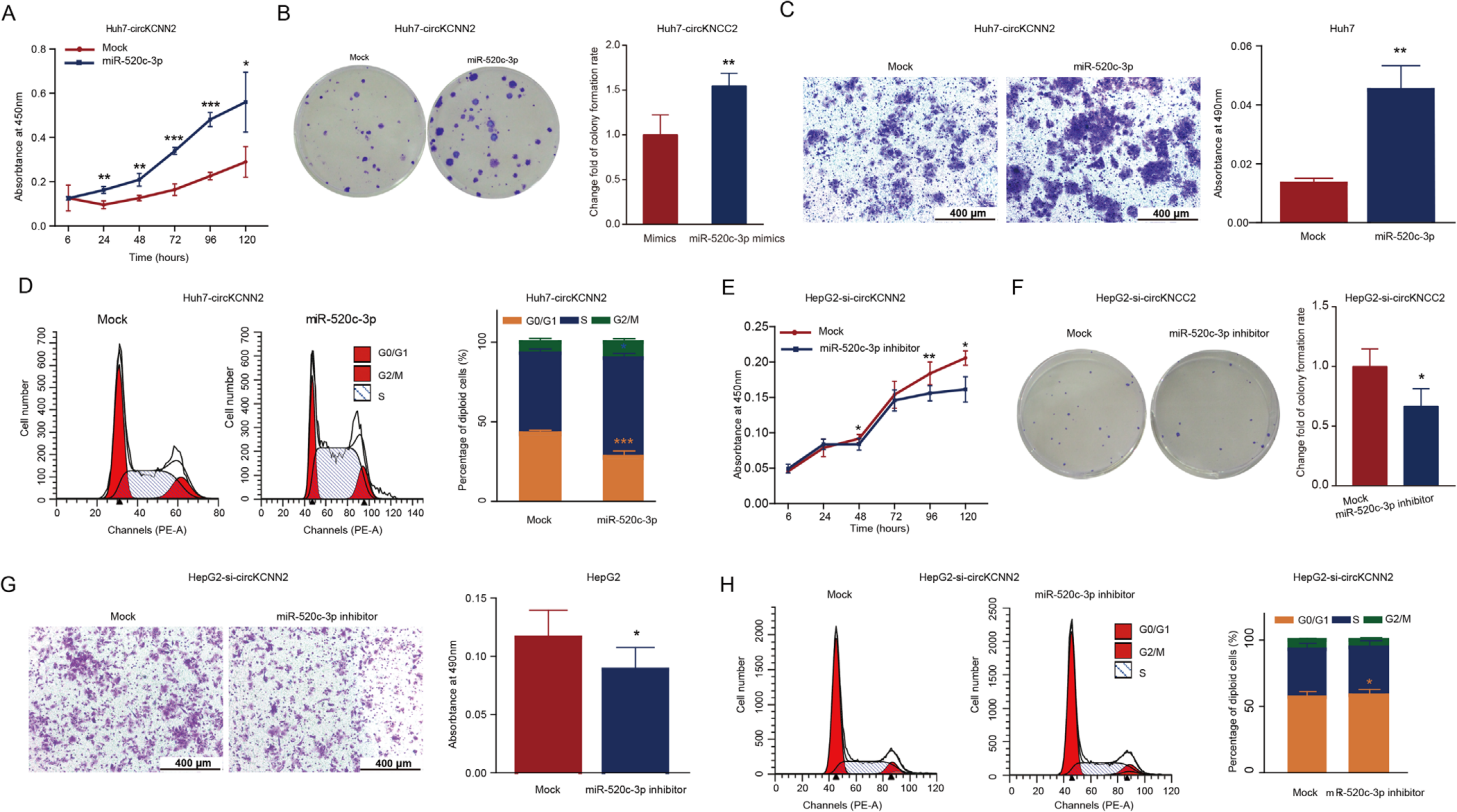
miR‐520c‐3p affected the antitumor function of circKCNN2. (A‐C) The effects of miR‐520c‐3p mimics on the proliferation, colony formation, and migration of Huh7‐circKCNN2 cells. (D) The proportion of Huh7‐circKCNN2 cells at different stages of the cell cycle. (E‐G) The effects of miR‐520c‐3p inhibitor on the proliferation, colony formation, and migration of HepG2‐si‐circKCNN2 cells. (H) The proportion of HepG2‐si‐circKCNN2 cells at different stages of the cell cycle. All the assays were performed at least three times. N = 3 for each group. *P < 0.05, **P < 0.01, ***P < 0.001

To figure out whether circKCNN2 regulates the biological activity of HCC cells via sponging miR‐520c‐3p, the miR‐520c‐3p mimics were transfected into Huh7 cells with circKCNN2 overexpression and the miR‐520c‐3p inhibitors were transfected into HepG2 cells with circKCNN2 knockdown, respectively. miR‐520c‐3p mimics significantly neutralized the inhibiting effects of circKCNN2 on the proliferation, colony formation, and migration of Huh7 cells (Figure 8A–C). miR‐520c‐3p mimics also neutralized the inhibitory effect of circKCNN2 on the S phase of Huh7 cells (Figure 8D). miR‐520c‐3p inhibitors significantly reversed the effects of circKCNN2 knockdown on the proliferation, colony formation, and migration of HepG2 cells (Figure 8E–G). The proportion at G_0_/G_1_ stage was higher in HepG2 cells with silenced circKCNN2 plus miR‐520c‐3p inhibitor than in those plus miR‐520c‐3p inhibitor negative control (Figure 8H). Collectively, these data support that circKCNN2 inhibits the proliferation, migration, and colony formation capacity of HCC cells via targeting miR‐520c‐3p.

### 3.6 miR‐520c‐3p functions in HCC cells partially via regulating MBD2

**FIGURE 9:**
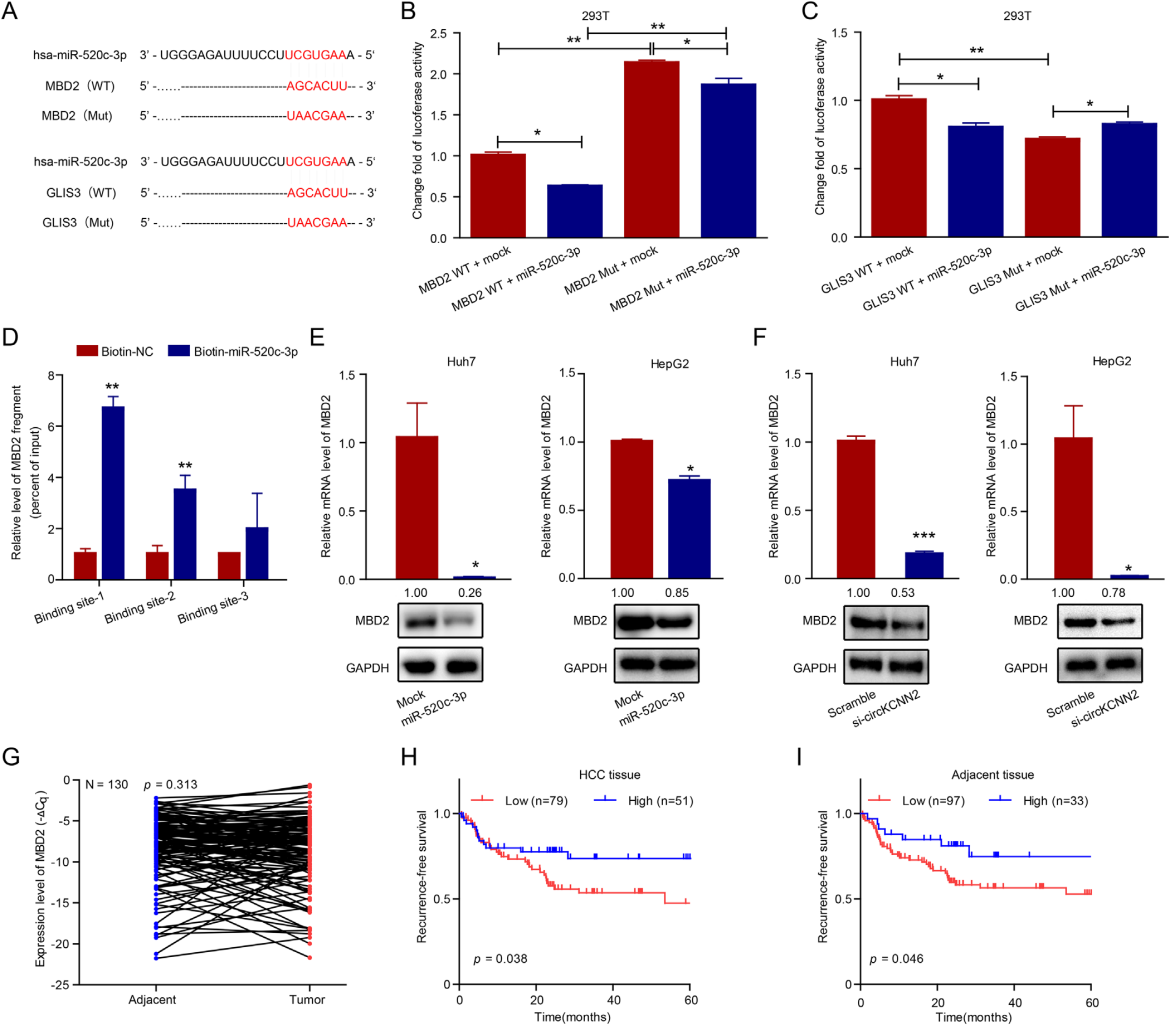
MBD2 was the target of miR‐520c‐3p in HCC cells. (A) The potential binding sites of miR‐520c‐3p with MBD2 and GLIS3. (B, C) The luciferase activity in 293T cells after co‐transfection with the reporter plasmids and miR‐520c‐3p mimics. Groups of MBD2 WT + mock and GLIS3 WT + mock served as references for normalization. (D) Results of RNA pull‐down followed RT‐qPCR testing the enrichment level of MBD2 3′UTR in the RNA pulled down by biotin‐miR‐520c‐3p probe or control probe (Biotin‐NC, reference for calculating relative value). Binding site 1–3, different region of MBD2 3′UTR that contains one of the three putative binding sites. (E) The effect of miR‐520c‐3p mimics on the mRNA and protein levels of MBD2 in Huh7 and HepG2 cells. Groups of mock served as references for calculating relative mRNA levels. (F) The effect of circKCNN2 knockdown on the mRNA and protein levels of MBD2 in Huh7 and HepG2 cells. Groups of si‐scramble served as references for calculating relative mRNA level. (G) The expression level of MBD2 in human HCC tumor tissues and adjacent tissues. (H) The correlation between the mRNA level of MDB2 in HCC tissue and the recurrence‐free survival of HCC. (I) The correlation between the mRNA level of MDB2 in adjacent tissue and the recurrence‐free survival of HCC. All the assays were performed at least three times. N = 3 for each group. *P < 0.05, **P < 0.01, ***P < 0.001

RT‐qPCR results showed that the ectopic expression of circKCNN2, knockdown of circKCNN2, miR‐520c‐3p mimics, and inhibitor of miR‐520c‐3p had no significant effect on the linear mRNA level of KCNN2 (Figure S4). circKCNN2 may inhibit HCC development by regulating the downstream molecules instead of its parental gene. It was reported that glypican 3 (GPC3) was the target of miR‐520c‐3p.^23^ However, our RT‐qPCR tests indicated that the expression level of GPC3 in Huh7 and HepG2 cells was not affected by the transfection with the inhibitor or mimics of miR‐520c‐3p (Figure S5). By querying the miRDIP database (https://www.biostars.org/p/317966/) and miRTarBase (https://maayanlab.cloud/Harmonizome/resource/MiRTarBase), three putative binding sites of miR‐520c‐3p were found at each of the 3′ untranslated regions (3′UTR) of MBD2 and GLIS3 (Figure 9A, Table S5). The three putative binding sites in the same gene have the same sequence. The luciferase activity in 293T cells transfected with MBD2‐WT was down‐regulated by 38.0% with miR‐520c‐3p mimics and could be restored by mutating the binding sites at the 3′UTR of MBD2. The luciferase activity in cells with MBD2‐Mut was also down‐regulated by 13.0% with miR‐520c‐3p mimics (Figure 9B), implying that other binding sites might exist in the mutated 3′UTR of MBD2. miR‐520c‐3p mimics inhibited luciferase activity in cells with GLIS3‐WT, but the mutations could not restore the luciferase activity (Figure 9C), indicating that GLIS3 was not the target of miR‐520c‐3p. RNA pull‐down assay was performed to confirm the functional binding region from three putative binding sites (reference, NM_333927.5; binding site 1, 1641 bp‐1648 bp; binding site 2, 4179 bp‐4186 bp; binding site 3, 4566 bp‐4572 bp). The RNA pull‐down followed RT‐qPCR showed that the fragments carrying binding site 1 or binding site 2 were significantly enriched in the RNA binding to miR‐520c‐3p (Figure 9D). Both the miR‐520c‐3p mimics and the knockdown of circKCNN2 significantly downregulated the mRNA and protein levels of MBD2 (Figure 9E, F). However, the levels of MBD2 mRNA between HCC tissues and paired adjacent liver tissues were not significantly different (Figure 9G). Our tumor transplantation experiment showed that no significant difference in the MBD2 protein level was observed in the tumors of the mice among different groups (Figure S6). However, high expression of MBD2 in HCC tissues and paired adjacent tissues predicted a favorable RFS (Figure 9H and I), but did not affect OS (Figure S7). These observations indicate that HCC progression is, at least partially, inhibited by circKCNN2 via regulating the miR‐520c‐3p/MBD2 axis.

### 3.7 The association between circKCNN2 and the therapeutic effect of lenvatinib

**FIGURE 10:**
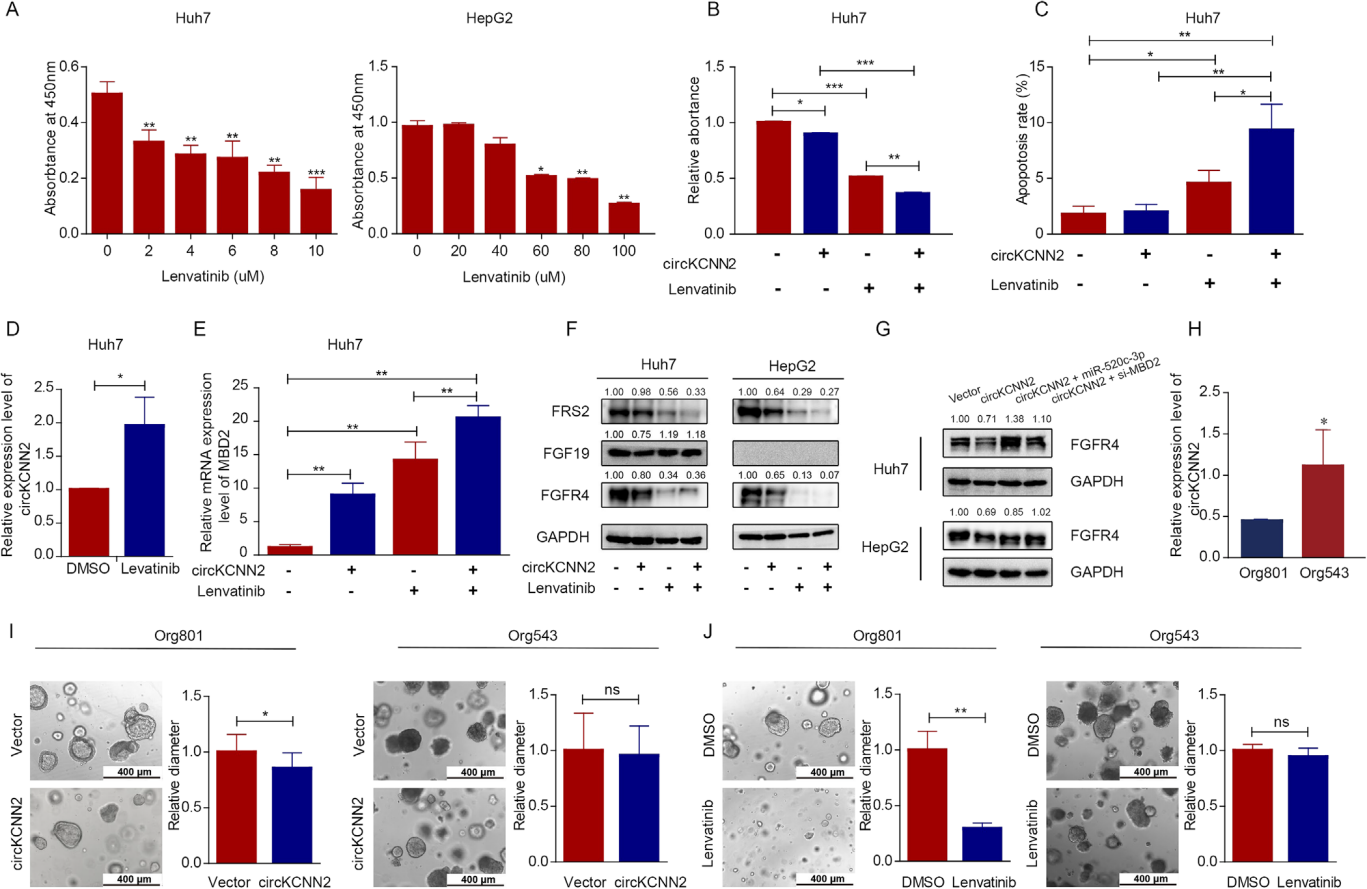
The association between circKCNN2 and the therapeutic effect of lenvatinib. (A) The proliferation of Huh7 and HepG2 cells were inhibited by different doses of lenvatinib. (B) The effect of lenvatinib treatment and circKCNN2 overexpression on the proliferation of Huh7 cells. (C) The effect of lenvatinib treatment and circKCNN2 overexpression on the apoptosis of Huh7 cells. (D) Lenvatinib treatment elevated the RNA level of circKCNN2 in Huh7 cells. The DMSO group served as a reference for normalization. (E) circKCNN2 and lenvatinib enhanced the expression of MBD2 in Huh7 cells. The group without treatment served as a reference for normalization. (F) The effect of circKCNN2 and lenvatinib on the protein levels of FRS2, FGF19, and FGFR4 in Huh7 and HepG2 cells. (G) The miR‐520c‐3p mimics and the knockdown of MBD2 can reverse the circKCNN2‐induced downregulation of FGFR4. (H) The inherent levels of circKCNN2 in the two HCC organoids. Org543 served as a reference for normalization. (I) The effect of circKCNN2 overexpression on the cell growth of HCC organoids. (J) The effect of lenvatinib on the cell growth of HCC organoids. All the assays were performed at least three times. n = 3 for each group. *P < 0.05, **P < 0.01, ***P < 0.001

We found that the cells with a low inherent level of circKCNN2 were more sensitive to the treatment of lenvatinib. As mentioned above, the baseline level of circKCNN2 was around 80‐fold higher in HepG2 cells than in Huh7 cells (Figure 3A left). The proliferation of Huh7 cells was significantly inhibited by lenvatinib at a low dose of 2μM. However, in HepG2 cells, the cell proliferation was inhibited by lenvatinib only at the dose higher than 60μM (Figure 10A). The half‐maximal inhibitory concentration (IC50) of lenvatinib was 8.14±0.12 μM and 57.47±0.92 μM in Huh7 cells and HepG2 cells, respectively. In the following experiments investigating the therapeutic effect of lenvatinib, Huh7 cells, HepG2 cells, and organoids were treated at a dose of 10μM, 60μM, and 35μM, respectively. Interestingly, ectopic overexpression of circKCNN2 by infection and lenvatinib had synergistic effects. The combination of lenvatinib and circKCNN2 overexpression showed the most powerful ability to inhibit the proliferation and promote the apoptosis in Huh7 and HepG2 cells, compared with each of them (Figure 10B, C, and Figure S8A–S8C). The treatment of lenvatinib significantly upregulated the expression of circKCNN2 and MBD2 (Figure 10D,E). Among all the targets of lenvatinib, FGFR4 is mostly expressed in liver tissue.^24^ Fibroblast growth factor 19 (FGF19) is the endogenous FGF member with the highest affinity to FGFR4.^25^ The activation of FGF19/FGFR4/fibroblast growth factor receptor substrate 2 (FRS2) pathway promotes HCC evolution.^25^ Ectopic overexpression of circKCNN2 significantly enhanced the inhibition of FRS2 by lenvatinib in both Huh7 and HepG2 cells (Figure 10F). However, the ectopic expression of circKCNN2 alone can only decrease the protein level of FRS2 in HepG2, but not in Huh7 cells. Notably, FGF19 expression was normal in Huh7 cells but was absent in HepG2 cells. In Huh7 cells, the level of FGF19 was decreased in the group with circKCNN2 overexpression and increased in the group with lenvatinib therapy. The mRNA level and protein level of FGFR4 were significantly inhibited by circKCNN2 in both HepG2 and Huh7 cells (Figure 10F and Figure S8D). The mimics of miR‐520c‐3p and knockdown of MBD2 significantly reversed the circKCNN2‐induced downregulation of FGFR4 (Figure 10G). Thus circKCNN2 inhibits the expression of FGFR4 through regulating miR‐520c‐3p/MBD2 axis.

The association between the therapeutic effect of lenvatinib and circKCNN2 expression was further confirmed in two human HCC organoid models, Org801 and Org543. We developed the two organoids from fresh HCC samples of patients with the same stage and similar tumor size (Table S9). The inherent level of circKCNN2 was approximately 1.5‐fold higher in Org543 than in Org801 (Figure 10H). To the last follow‐up day (March 20, 2021), the HCC patient from which Org543 was derived had no recurrence within 30 months after surgery. The HCC patient from which Org801 was derived had an early relapse at the 4th month after surgery. The growth of Org801 was suppressed by ectopic overexpression of circKCNN2 and significantly suppressed by lenvatinib (Figure 10I, J, S8E). Both circKCNN2 and lenvatinib had no therapeutic effect on Org543.

## 4 DISCUSSION

In this study, circKCNN2 was first identified as a predictive biomarker of HCC recurrence. The circKCNN2 level was lower in the HCC tissues than in the adjacent tissues. The level of circKCNN2 was also lower in tumor tissues from recurrent patients than in those of non‐recurrent patients (Figure 1A). Furthermore, the low level of circKCNN2 was significantly associated with unfavorable OS and RFS of HCC. circKCNN2 was previously reported to be associated with the development of glioma.^22^ These data, together with our results, indicate that circKCNN2 may function as a tumor suppressor in multiple cancer types. Although the statistical association between circKCNN2 and cancer progression has been reported previously, the underlying mechanisms remain largely unknown.

This study firstly validated the back‐splicing junction site of circKCNN2. The significant association between the expression of circKCNN2 and linear KCNN2 was revealed, suggesting that circKCNN2 was derived from linear KCNN2 in a fixed fashion. The role of KCNN2, the Ca^2+^‐dependent potassium channel, in the development of malignant diseases has not been elucidated. KCNN2 significantly decreased in HCC tissues, suggesting the parental gene of circKCNN2 may also act as a tumor suppressor in HCC. The expression of KCNN2 and circKCNN2 was under the transcriptional regulation of NFYA. The high level of NFYA was significantly associated with an increased risk of postoperative HCC recurrence. NFYA is a transcription factor that was reported to promote the development of many cancers, including HCC. It was reported that NFYA was upregulated in cancer stem cells of HCC.^26^ NFYA also contributes to HCC by enhancing the generation of a tumor‐specific transcript of *lin‐28 homolog B* (*ILN28*), a gene highly expressed during embryogenesis but silent after birth.^27^ NFYA also inhibits the tumor suppressor function in lung cancer through recruiting the transcriptional repressor to the promoter of a tumor suppressor.^28^ Demethylation of promoters contributes to the recruitment and binding of NFYA.^26^, ^29^ Thus, the binding between NFYA and the promoter of *KCNN2* may be enhanced by demethylation, resulting in a decrease in the translation of *KCNN2* gene. The down‐regulation of *KCNN2* transcription leads to reduced expression of circKCNN2, which facilitates the progression of HCC.

We found that circKCNN2 attenuated the proliferation, migration, and colony formation of HCC cells (Figure 2). miR‐520c‐3p was an important target of circKCNN2 and reversed the effects of circKCNN2 on HCC cells. Interestingly, miR‐520c‐3p was reported to enhance the chemo‐sensitivity of HepG2 cells to doxorubicin treatment.^30^ This may be due to the fact that miR‐520c‐3p increases the proportion of cells at the S stage (Figure 7). miR‐520c‐3p was significantly upregulated in some cancers including early‐stage non‐small‐cell lung cancer and gastric cancer,^31^, ^32^ and significantly associated with the stemness and metastasis in gastric cancer via inducing epithelial‐to‐mesenchymal transition.^32^ The data in this study and another study support that miR‐520c‐3p promotes the progression of HCC.^33^ However, miR‐520c‐3p also functions as a tumor suppresser in lymphoma.^34^ These seemingly controversial data on the function of miR‐520c‐3p indicate that miR‐520c‐3p is a mediator involved in complex signaling cascades. A study of testicular germ cell tumor has demonstrated that miR‐520c‐3p is the hub molecule of the network regulating gene expression and DNA methylation.^35^ Consistent with this evidence, we identified a target of miR‐520c‐3p, MBD2, a key molecule hub playing an important role in methylation‐related transcription regulation.

MBD2, a methyl‐CpG binding protein, binds a significant fraction of the hypomethylated genes, determines RNA polymerase II binding and DNA methylation state.^36^ The role of MBD2 in carcinogenesis is not consistent, possibly because MBD2 has different alternative splicing variants. MBD2a promotes, whereas its short form MBD2c suppresses, cancer metastasis.^37^ There is no study reporting alternative splicing variants of MBD2 in the liver. Our study found that the high level of MBD2 in tumor and adjacent tissue predicted a favorable RFS, indicating the variant of MBD2 is likely to be MBD2c. This effect of MBD2 on HCC recurrence is consistent with that of circKCNN2. MBD2 has been proven to significantly inhibit the expression of telomerase reverse transcriptase (TERT) via binding the hypermethylated region of the *TERT* promoter in liver, breast, cervical, and neuroblastoma cell lines.^38^ TERT is essential for the maintenance of telomere length, thus influencing cellular immortality. Therefore, MBD2 may be a common suppressor for several cancer types, which is similar to circKCNN2. It has been revealed that global enhancer hypomethylation occurs in human HCCs,^39^ which may attenuate the inhibiting effect of MBD2 on functional oncogenes. Systemic inflammation is an important indicator for unfavorable postoperative prognosis in HCC.^40^ MBD2 mediates epigenetic transcriptional silencing by binding to methylated DNA, thus playing an important role in preventing tumor‐promoting inflammation.^41^ In our validation cohort, the levels of MBD2 in tumor and adjacent tissues were significantly associated with the postoperative survival of HCC although no significant difference was observed in the level of MBD2 between tumor tissues and adjacent tissues. Thus, MBD2 may play an important role at the late phase of HCC development rather than at the early phase.

To our knowledge, this is the first study reporting the association between circRNA and the FGF19/FGFR4/FRS2 pathway, which affects the therapeutic effect of lenvatinib. circKCNN2 down‐regulates FGF19 in Huh7. The high level of inherent circKCNN2 and the absence of FGF19 were observed in HepG2 cells. The expression of FGF19 is reported to be suppressed by proteins of the MBD family.^42^ Both circKCNN2 and lenvatinib might downregulate the expression of FGFR4 and FRS2. Ectopic overexpression of circKCNN2 enhanced the therapeutic effect of lenvatinib. Compared to ectopic expression of circKCNN2, lenvatinib was more efficient in reducing the expression of FGFR4 and FRS2. Lenvatinib also upregulated the expression of circKCNN2 and MBD2. Therefore, up‐regulation of circKCNN2 may be one of the mechanisms by which lenvatinib exerts its antitumor effect. circKCNN2 downregulates FGFR4 through the miR‐520c‐3p/MBD2 axis. Thus, the effectiveness of lenvatinib may be reduced because the FGF19/FGFR4/FRS2 pathway is already inhibited by the high inherent level of circKCNN2. Inherent level of circKCNN2 in HCC cells predisposes anti‐tumor effect of lenvatinib possibly because circKCNN2 and lenvatinib repress the expression of FGFR4.

Our study has limitations. First, only one functional axis of circKCNN2 was systemically investigated, which might be not enough to comprehensively reveal the mechanism underlying the effect of circKCNN2 on HCC. Second, the association between epigenetic modification and transcription of circKCNN2 remains to be further investigated. Third, the effect of inherent circKCNN2 level on lenvatinib resistance was only investigated in two organoid models and two cell lines. The influence of potential confounding factors could not be excluded from the present study. Further epidemiological and molecular studies are needed to explore the underlying mechanisms of these findings.

Conclusively, we, for the first time, revealed that circKCNN2, whose transcription is repressed by NFYA, suppresses HCC recurrence via the miR‐520c‐3p/MBD2 axis. Inherent level of circKCNN2 in HCC cells predisposes anti‐tumor effect of lenvatinib possibly because circKCNN2 and lenvatinib repress the expression of FGFR4. circKCNN2 may be a promising predictive biomarker and therapeutic agent for the treatment of HCC recurrence.

## CONFLICT OF INTEREST

The authors have declared that no conflict of interest.

## Supporting information

Supporting information
