# Supplementary material for: circKCNN2 suppresses the recurrence of hepatocellular carcinoma at least partially via regulating miR‐520c‐3p/methyl‐DNA‐binding domain protein 2 axis
Source: Clin Transl Med. 2022 Jan 20;12(1):e662. doi: 10.1002/ctm2.662 (PMC8775140; doi:10.1002/ctm2.662)

Supplementary Figure S6

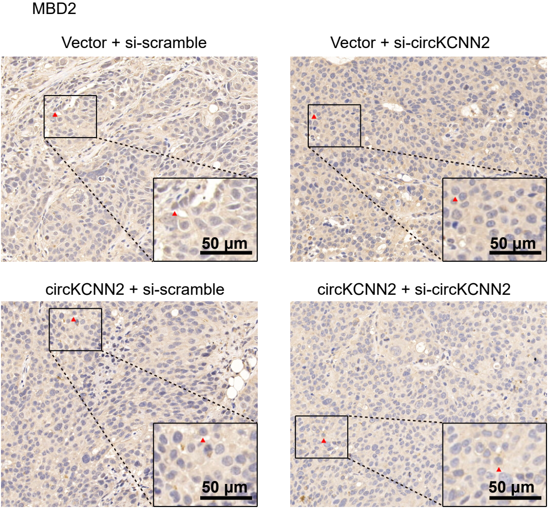

Supplement: Supplementary file 1 — Supporting information [file CTM2-12-e662-s001.zip › Supplementary Figure S6.pdf]
