# Supplementary material for: circKCNN2 suppresses the recurrence of hepatocellular carcinoma at least partially via regulating miR‐520c‐3p/methyl‐DNA‐binding domain protein 2 axis
Source: Clin Transl Med. 2022 Jan 20;12(1):e662. doi: 10.1002/ctm2.662 (PMC8775140; doi:10.1002/ctm2.662)
Supplement: Supplementary file 1 — Supporting information [file CTM2-12-e662-s001.zip › Supplemetary material.docx]

**Supplementary methods**

**Differential expression analysis and gene set enrichment analysis (GSEA)**

The circRNAs were identified by CIRI software ^[1]^. The gene expression was evaluated by eXpress on the reference transcriptome (GRCh38) ^[2]^. Differential expression analyses of circRNAs and genes were performed using the edgeR package ^[3]^. The circRNAs and genes with fold change (FC) ≥ 2 and false discover rate (FDR) < 0.05 were defined as differentially expressed. Taking each differentially expressed circRNA as a profile, we applied GSEA with the “Prerank” strategy ^[4]^. The phenotype was permutated 1,000 times and the gene sets with an FDR less than 0.25 were considered significantly enriched.

**Construction of lentivirus**

The sequence of circKCNN2 was synthesized by Obio Technology (Shanghai, China) and was cloned into the H8399 pLenti-EF1a-EGFP-F2A (Obio Technology), a circRNA overexpression vector that can induce the circularization of the targeting sequence. The constructed lentiviral vectors were transfected into HEK293T cells together with the MD2G packaging plasmid and PAX2 envelope plasmid by using Lipofectamine 3000 kit (Invitrogen). After incubation for 48h, lentivirus-containing supernatants were harvested and lentiviral particles were concentrated using Lenti-X (Clontech, CA). Huh7 and HepG2 cells were infected with lentivirus for 24 h and the stable cell strains were screened by 2 μg/mL puromycin dihydrochloride (Thermo Fisher, Waltham, MA). The overexpression and circularization of circKCNN2 were confirmed by RT-qPCR with primers covering the junction site.

**Cell culture**

Human HCC cell lines (Huh7 and HepG2) and HEK293T cell lines were purchased from the Chinese Academy of Sciences (Shanghai, China). All cells were cultured in Dulbecco’s modified Eagle’s medium (DMEM) (HyClone, Logan, UT) with 10% fetal bovine serum (FBS) (Gibco, New York, NY), 1% penicillin and streptomycin (Bio-light, Shanghai, China) at 37ºC and 5% CO_2_. Small interfering RNAs (siRNAs) against circKCNN2 were designed and synthesized by GenePharma (Shanghai, China). The mimics and inhibitors of miR-520c-3p were purchased from GenePharma. Lipofectamine 3000 kit (Invitrogen, Carlsbad, CA) was applied to transfect the siRNAs, miRNA mimics, miRNA inhibitors, and corresponding control oligonucleotides into cells. Huh7 and HepG2 cells were infected with lentivirus for 24 h and the stable cell strains were screened by 2 μg/mL puromycin dihydrochloride (Thermo Fisher, Waltham, MA). Organoids were digested into single cells and incubated with lentivirus for 10min, then organoids were re-plated into plates. Infected organoids were selected from 3 days post-infection for 1 week with 2.5 μg/mL puromycin in order to remove uninfected organoids

**Cell proliferation, colony formation, and migration assay**

Cell proliferation was assessed by the Cell Counting Kit-8 (CCK8) kit (Dojindo, Osaka, Japan). Cells were seeded into the 96-well plates (Corning Incorporated Coster, Kennebunk, ME). Then, 100uL of 10% CCK8-DMEM solution was added and the cells were incubated for 1.5 h. The number of cells was estimated by measuring optical density (OD450) for every 24 h. For colony formation assay, 500 cells were seeded into 6-well plates (Corning) which contain 500 μL DMEM (10% FBS). Then cells were incubated for 21 days. Cell clones were stained with crystal violet and counted manually. The experiments were performed in triplicate.

The ability of cell migration was measured with Transwell inserts (Corning, New York, NY). A total of 1×10^5^ cells were placed in upper wells with 400µL serum-free DMEM containing 0.1% BSA (Bio-light). The lower chamber was filled with 500 µL DMEM (10% FBS). After incubation for 48 h, the migration cells were digested and seeded into 96-wells plates. After incubation for 6 h, 100µL DMEM containing 20% 3-(4,5dimethylthiazol-2-yl)-5-(3-carboxymethoxyphenyl)-2-(4-sulfophenyl)- 2H-tetrazolium inner salt (MTS) (Promega, Madison, WI) and phenazine methosulfate (PMS) (Promega, Madison, WI ) was added. After 2 h incubation, absorbance at 490 nm was measured. Five fields were randomly selected and photographed with a microscope at 10 × magnification. Each assay was performed in triplicate.

**Reference:**

1. Gao Y, Wang JF, Zhao FQ. CIRI: an efficient and unbiased algorithm for de novo circular RNA identification. *Genome Biol*. 2015; 16:4.

2. Roberts A, Pachter L. Streaming fragment assignment for real-time analysis of sequencing experiments. *Nat Methods*. 2013; 10: 71-73.

3. McCarthy DJ, Chen Y, Smyth GK. Differential expression analysis of multifactor RNA-Seq experiments with respect to biological variation. *Nucleic Acids Res*. 2012; 40: 4288-4297.

4. Subramanian A, Tamayo P, Mootha VK, et al. Gene set enrichment analysis: a knowledge-based approach for interpreting genome-wide expression profiles. *Proc Natl Acad Sci U S A*. 2005; 102: 15545-15550.

**Supplementary figure legend**

**Figure S1. The identification of the functional transcription factor regulating circKCNN2.** (A) The expression levels of putative transcriptional factors in the tumor and adjacent tissues of the 13 HCC patients from training cohort. (B) The expression levels of putative transcriptional factors in the tumor and adjacent tissues of HCC patients from TCGA LIHC cohort. (C) The association between the intratumoral expression of putative transcriptional factors and the recurrence-free survival of HCC. (D) Results of ChIP followed qPCR testing the enrichment level of KCNN2 promoter. IgG, DNA pulled down by IgG; JUNB, DNA pulled down by the antibody of JUNB. The group of IgG served as a reference to calculate a relative enrichment. (E) RT-qPCR results displaying the levels of *KCNN2* and circKCNN2. The groups of si-scramble served as references to calculate relative RNA levels. Cellular experiments were performed in triplicate. n=3 for each group. ns, no statistically significant; *, *P* <0.05; **, *P* <0.01; ***, *P* <0.001.

**Figure S2. The expression level of miR-520c-3p in the tumor and adjacent tissues from 130 HCC patients of the validation cohort.** (A) RT-qPCR showed that the level of miR-520c-3p was significantly higher in tumor tissues than paired adjacent tissues. (B) The intratumoral expression of miR-520c-3p was significantly higher in patients with recurrence, compared with that without recurrence. *, *p<*0.05; **, *p<*0.01..

**Figure S3. The efficiency of miR-520c-3p inhibitor and mimics.** (A) The efficiency of miR-520c-3p mimics in Huh7 cells. The level of miR-520c-3p was investigated by RT-qPCR. (B) The efficiency of miR-520c-3p mimics in HepG2 cells. (C) The efficiency of miR-520c-3p inhibitor in Huh7 cells. (D) The efficiency of miR-520c-3p inhibitor in HepG2 cells.

**Figure S4. The levels of KCNN2 in cells with altered levels of circKCNN2 or miR-520c-3p.** (A) The levels of KCNN2 in cells with altered levels of circKCNN2. Scramble, si-circKCNN2, vector, and circKCNN2: cells transfected with siRNA negative control, siRNA targeting circKCNN2, empty vector plasmid, overexpression plasmid of circKCNN2. (B) The levels of KCNN2 in cells with altered levels of miR-520c-3p. NC, inhibitor, mock, mimics: cells treated with miRNA inhibitor control, inhibitor of miR-520c-3p, miRNA mimics control, mimics of miR-520c-3p.

**Figure S5. The mRNA expression of GPC3 in Huh7 and HepG2 cells.** (A) The mRNA level of GPC3 in Huh7 cells after the transfection of miR-520c-3p inhibitor. (B) The mRNA level of GPC3 in HepG2 cells after the transfection of miR-520c-3p inhibitor. (C) The mRNA level of GPC3 in Huh7 cells after the transfection of miR-520c-3p mimics. (D) The mRNA level of GPC3 in HepG2 cells after the transfection of miR-520c-3p mimics.

**Figure S6. The expression of MBD2 in mice xenograft.** The expression of MBD2 was stained by IHC.

**Figure S7. The association between overall survival and MBD2 level in HCC and paired adjacent tissues.** (A) The overall survival of HCC patients with different levels of MBD2 in tumor tissue. (B) The overall survival of HCC patients with different levels of MBD2 in adjacent tissue.

**Figure S8. The association between circKCNN2 and the therapeutic effect of lenvatinib.** (A) The effect of lenvatinib treatment and circKCNN2 overexpression on the proliferation of HepG2 cells. (B) The effect of lenvatinib treatment and circKCNN2 overexpression on the apoptosis of HepG2 cells. (C) Representative flow cytometry plots for the apoptosis analysis of Huh7 cells with different treatments. (D) The effect of circKCNN2 overexpression on the mRNA level of FGR4, FGF19, and FRS2. (E) HCC organoids were successfully infected with circKCNN2 overexpression lentivirus. The cells were infected by the lentivirus expressing GFP. The transfection efficiency was assessed by in situ GFP expression. The efficiency of circKCNN2 was confirmed by RT-qPCR. * *p* <0.05, ** *p* <0.01, *** *p* <0.001.

**Supplementary Table S1. The clinical characteristics of 13 HCC patients in the investigation cohort.**

| Clinical characteristics | | without recurrence | with recurrence | *P*-value |
| --- | --- | --- | --- | --- |
|  |  | (n = 9) | (n = 4) |  |
| Male |  | 7 | 3 | 0.98 |
| Age | <55 years | 3 | 3 | 0.96 |
|  | ≥55 years | 6 | 1 |  |
| Fibrosis | Yes | 5 | 1 | 0.87 |
|  | No | 4 | 3 |  |
| MVI | Yes | 7 | 3 | 0.98 |
|  | No | 2 | 1 | 0.86 |
| BCLC | 0/A | 8 | 4 |  |
|  | B | 1 | 0 |  |
| Envelope | Yes | 8 | 2 | 0.28 |
|  | No | 1 | 2 |  |
| HBsAg | negative | 0 | 1 | 0.12 |
|  | positive | 9 | 3 |  |
| AST(U/L) |  | 33.9(28.5-44.1) | 25.4(22.4-31.5) | 0.15 |
| ALT(U/L) |  | 41.4(26.6-39.4) | 28.7(20.9-39.8) | 0.52 |
| AFP(U/L) |  | 203.8(113.6-1210.0) | 146.6(16.8-466) | 0.59 |
|  |  |  |  |  |

AST: aspartate aminotransferase; ALT: alanine aminotransferase; AFP: α-fetoprotein; MVI: microscopic vascular invasion; BCLC: Barcelona Clinic Liver Cancer.

**Supplementary Table S2. The clinical characteristics of 130 HCC patients in the validation cohort.**

| Clinical characteristics |  | Total patients | without recurrence | with recurrence | *P*-value |
| --- | --- | --- | --- | --- | --- |
|  |  | (N = 130) | (n = 84) | (n = 46) |  |
| Male |  | 110 | 70 | 40 | 0.58 |
| Age | <55 years | 60 | 40 | 20 | 0.65 |
|  | ≥55 years | 70 | 44 | 26 |  |
| Fibrosis | Yes | 77 | 51 | 26 | 0.64 |
|  | No | 53 | 33 | 20 |  |
| MVI | Yes | 90 | 61 | 29 | 0.26 |
|  | No | 40 | 23 | 17 |  |
| BCLC | 0/A | 32 | 23 | 9 | 0.32 |
|  | B | 98 | 61 | 37 |  |
| Envelope | Yes | 11 | 8 | 3 | 0.56 |
|  | No | 119 | 76 | 43 |  |
| HBsAg | negative | 18 | 10 | 8 | 0.39 |
|  | positive | 112 | 74 | 38 |  |
| HBV DNA | <500 ng/mL | 82 | 45 | 27 | 0.57 |
|  | ≥500 ng/mL | 58 | 39 | 19 |  |
| Tumor size | <5 cm | 24 | 19 | 5 | 0.10 |
|  | ≥5 cm | 106 | 65 | 41 |  |
| AFP | <400 ng/mL | 76 | 52 | 24 | 0.28 |
|  | ≥400 ng/mL | 54 | 32 | 22 |  |
| circKCNN2 | Low^*^ | 97 | 39 | 30 | **0.04** |
|  | High^#^ | 33 | 45 | 16 |  |

MVI: microscopic vascular invasion; BCLC: Barcelona Clinic Liver Cancer; AFP: α-fetoprotein; ^*^ Patients that had a lower circKCNN2 expression level than that averge expression lever in our HCC tissues; ^#^ Patients that had a higher circKCNN2 expression level than that averge expression level in our HCC tissues.

**Supplementary Table S3. The sequences of siRNAs.**

| **Name** | **Sequence** |  |
| --- | --- | --- |
| si-circKCNN2-1 | Sense (5’-3’) | CCGAGCUUGUGAAAGUUGUTT |
|  | Antisense (5’-3’) | ACAACUUUCACAAGCUCGGTT |
| si-circKCNN2-2 | Sense (5’-3’) | GCUUGUGAAAGUUGUUCAUTT |
|  | Antisense (5’-3’) | AUGAACAACUUUCACAAGCTT |
| siRNA scramble | Sense (5’-3’) | UUCUCCGAACGUGUCACGUTT |
|  | Antisense (5’-3’) | ACGUGACACGUUCGGAGAATT |
| si-JUNB | Sense (5’-3’) | ACAAGGUGAAGACGCUCAATT |
|  | Antisense (5’-3’) | UUGAGCGUCUUCACCUUGUTT |
| si-NFYA | Sense (5’-3’) | GUGCCUGCUAUCCAAAGAATT |
|  | Antisense (5’-3’) | UUCUUUGGAUAGCAGGCACTT |
| si-MBD2 | Sense (5’-3’) | GCAAGAGCGATGTCTACTA |
|  | Antisense (5’-3’) | CGTTCTCGCTACAGATGAT |

**Supplementary Table S4. The mimics and inhibitors of miR-520c-3p.**

| **Name** | **Sequence** |  |
| --- | --- | --- |
| miR-520c-3p mimics | Sense (5’-3’) | AAAGUGCUUCCUUUUAGAGGGU |
|  | Antisense (5’-3’) | CCUCUAAAAGGAAGCACUUUUU |
| Mock for mimics | Sense (5’-3’) | UUCUCCGAACGUGUCACGUTT |
|  | Antisense (5’-3’) | ACGUGACACGUUCGGAGAATT |
| miR-520c-3p inhibitors | Sense (5’-3’) | ACCCUCUAAAAGGAAGCACUUU |
| Mock for inhibitors | Sense (5’-3’) | CAGUACUUUUGUGUAGUACAA |

**Supplementary Table S5. Sequences of circKCNN2, MBD2 3’UTR, and GLIS3 3’UTR cloned into the pMIR-REPORT luciferase vector.**

| **Name** | **Sequences** |
| --- | --- |
| **circKCNN2 WT** | TTGTTCATGGTGGACAATGGAGCAGATGACTGGAGAATAGCCATGACTTATGAGCGTATTTTCTTCATCTGCTTGGAAATACTGGTGTGTGCTATTCATCCCATACCTGGGAATTATACATTCACATGGACGGCCCGGCTTGCCTTCTCCTATGCCCCATCCACAACCACCGCTGATGTGGATATTATTTTATCTATACCAATGTTCTTAAGACTCTATCTGATTGCCAGAGTCATGCTTTTACATAGCAAACTTTTCACTGATGC**CTCCTCTAGAAGCATTGGAGCACTTA**ATAAGATAAACTTCAATACACGTTTTGTTATGAAGACTTTAATGACTATATGCCCAGGAACTGTACTCTTGGTTTTTAGTATCTCATTATGGATAATTGCCGCATGGACTGTCCGAGCTTGTGAAAG |
| **circKCNN2 Mut** | TTGTTCATGGTGGACAATGGAGCAGATGACTGGAGAATAGCCATGACTTATGAGCGTATTTTCTTCATCTGCTTGGAAATACTGGTGTGTGCTATTCATCCCATACCTGGGAATTATACATTCACATGGACGGCCCGGCTTGCCTTCTCCTATGCCCCATCCACAACCACCGCTGATGTGGATATTATTTTATCTATACCAATGTTCTTAAGACTCTATCTGATTGCCAGAGTCATGCTTTTACATAGCAAACTTTTCACTGATGCATAAGATAAACTTCAATACACGTTTTGTTATGAAGACTTTAATGACTATATGCCCAGGAACTGTACTCTTGGTTTTTAGTATCTCATTATGGATAATTGCCGCATGGACTGTCCGAGCTTGTGAAAG |
| **MBD2 3’ UTR WT** | gaatatgatcaggtaactttcgaccgactttccccaagagaaaattcctagaaattgaacaaaaatgtttccactggcttttgcctgtaagaaaaaaaatgtacccgagcacatagagctttttaatagcactaaccaatgcctttttagatgtatttttgatgtatatatctattattcaaaaaatcatgtttattttgagtcctaggacttaaaattagtcttttgtaatatcaagcaggaccctaagatgaagctgagcttttgatgccaggtgcaatctactggaaatgt**agcactta^1^**cgtaaaacatttgtttcccccacagttttaataagaacagatcaggaattctaaataaatttcccagttaaagattattgtgacttcactgtatataaacatatttttatactttattgaaaggggacacctgtacattcttccatcatcactgtaaagacaaataaatgattatattcacagactgattggaattctttctgttgaaaagcacacacaataaagaacccctcgttagccttcctctgatttacattcaactctgatccctgggccttaggtttgacatggaggtggaggaagatagcgcatatatttgcagtatgaactattgcctctggacgttgtgagaattgtgctttcaccagaatttctaagaatttctgctaaatatcacctagcatgtgtaattttttttccttgcctgtgacttggacttttgatagttctataagaataaggctttttcttcccttgggcatgagtcagatacacaaggacccttcaggtgttactagaaggcgtccatgtttattgttttttaaagaatgtttggcactctctaacgtccactagcttactgagttatcaggtgcaggtcagactcttggctacagtgagaggcagcttctagacagagttgcttaatgaaagggtttgtaatactttacaaaccattacctgtacctggcctggcctccaaaatattaacattctttttctgttgaaactcgcgagtgtaactttcataccacttgaatttattgatatttaattatgaaaactagcattacattattaaacgatttctaaaatcaaaacatacttaatctgataccaaggaagggagggagtggttataagcaaatgaaaacaaattttgagagacagagcaaaagtaaaatcattctatagaaaaggtgtgtttatttcttaactcttgagttcttttaaaattagaacctaaatgatgcagtcaggggtatgaccaattccacatgagtgtcacctgtacattttattaccactacctccagtttccaaggcagggaagaagaggggaatagtcaaagcaatatgactagctaggtttgggctgctgtttgggctgctgtttttcctggacttttatgccaaactacataaatacttcttggaattcctgatttacgctcaactttgatccctggaccctagggccattagtttccttgaagcagtcctgctggtggatgagcagggaaacttgtagaccctgggaagccaattggtagagggtgcctgcccgccccatcagactgtcccaactggggtgggggtgaggatcatagtgatttctttttaaaagctagtatgactagtgagaatggatgtccaagggcttttctcttcctcccccgagtccacgattcttcatttgtgatcagggttggggtaacttcaccttggtgatcatatattctttttacaaaccaatccagccaagatatatgtgcttttctaaacagcttgtaaaagcaaaaacacaatgtatacacatataaaactgatttttttttttttgcttatatactttgctcaggtggagaacaaggtatgaaagccattatatggtgtcccttggggaccatcccataagtccagggtgttcccaatatacccacaagacaacatgttcaaaacttcatcacctggtccccacaaacctaccaccctcttccattccctcactctggtaaaagcaagccatcttctccagttacgcaaggcagaagcccaggagtgagcacaatgctgccctttcccttgcttctgacttctcatcaatcctattagaactactgactgcaccccacctctcctccatccccacgatcactgccttcatatgaactgcctttcctgtcctctggactttcagtggcttccttttttgctcacctgtccactttcttccccttccctctctccatccctccacatagtcatcagaaagatcatcaaaacaggcaaatgtgactgtattatttccctcacttatctttgtatgcagtttagagtgatttttaagagcacacactttgaaattaggagaacctgtgtctgaatcctgatttttgccatttgtgtgacttgtcaggttatttaatctctttgagcctgtccctcctcagtgaaataggaataataatacctacctcatagagttgttgtgaggattcagtgagatagactgtttctaaagtgtgtagcacagtgcctggcacatagtgggtgctcacattggagcacactattgtcgctatccctgcaggtgtgagttttacatcctttccaagaggcatttgctaataaccctaactaccgcctatccctgtgacactattttctgcctcagcaccctatttcttttgtagacattttctaagtctgtattttatttatttgttggctttttgtctatttctctactagaatgtaagctccatgagagcaaggatgtgtttgtctgatttcccgttacagcccc**agcactta^2^**gctgagtgactggcacctagtaagcactcagtaactgtttgctgaataaatggaagaatggtcaaaaggctcttcttgatctgatccctgcttgtctgtctggcctcatgctgccactattcctcactgcaccttcagctcaagaaaaacctctttacttgcttctttgaagtgccccttgggcctctgtgtctttgtacctgttgttccctttgcctgaaacaccctttgcctgcccgtctacatggtgaattcctaatcattgatgaagtctcagctcaatggctacctcctctgtgaagctgtccctgatttcttcaggcagatgttctttcccgccttccctgttcccaccgcactttgtaaatacctccatgga**agcactt^3^**tattgtattgtattgtaatttttttttgcatgtctgtttctactattagactgtaagcaccgtaaaagcagagattgtgctttttcatcctcatgttccccgagcctagcccagtgcctggcacgtaatagcttgtcattaaatatttgttgaatgaatctgtgaatgaacaagagtcatgcagttggaaaagtgttagaagctgtatgtattataaaggaaagaaatgcccttggtggcttccagggtgtttttttttttttttttggtctaaagaggagatctagagagtgttatacaatctctttgaatataagattactcgtttggatttttaaaataatatagatcttcataaataattgcaaagatataaacaagctttgtgatttctcaaatgctatgaagtccaaaataaatatttgcactattagtatttccatagtaaatgctatgagaaaatgtacagcaataaattttgaagctttaaaa |
| **MBD2 3’ UTR Mut** | gaatatgatcaggtaactttcgaccgactttccccaagagaaaattcctagaaattgaacaaaaatgtttccactggcttttgcctgtaagaaaaaaaatgtacccgagcacatagagctttttaatagcactaaccaatgcctttttagatgtatttttgatgtatatatctattattcaaaaaatcatgtttattttgagtcctaggacttaaaattagtcttttgtaatatcaagcaggaccctaagatgaagctgagcttttgatgccaggtgcaatctactggaaatgt**TAACGAA^1^**Ccgtaaaacatttgtttcccccacagttttaataagaacagatcaggaattctaaataaatttcccagttaaagattattgtgacttcactgtatataaacatatttttatactttattgaaaggggacacctgtacattcttccatcatcactgtaaagacaaataaatgattatattcacagactgattggaattctttctgttgaaaagcacacacaataaagaacccctcgttagccttcctctgatttacattcaactctgatccctgggccttaggtttgacatggaggtggaggaagatagcgcatatatttgcagtatgaactattgcctctggacgttgtgagaattgtgctttcaccagaatttctaagaatttctgctaaatatcacctagcatgtgtaattttttttccttgcctgtgacttggacttttgatagttctataagaataaggctttttcttcccttgggcatgagtcagatacacaaggacccttcaggtgttactagaaggcgtccatgtttattgttttttaaagaatgtttggcactctctaacgtccactagcttactgagttatcaggtgcaggtcagactcttggctacagtgagaggcagcttctagacagagttgcttaatgaaagggtttgtaatactttacaaaccattacctgtacctggcctggcctccaaaatattaacattctttttctgttgaaactcgcgagtgtaactttcataccacttgaatttattgatatttaattatgaaaactagcattacattattaaacgatttctaaaatcaaaacatacttaatctgataccaaggaagggagggagtggttataagcaaatgaaaacaaattttgagagacagagcaaaagtaaaatcattctatagaaaaggtgtgtttatttcttaactcttgagttcttttaaaattagaacctaaatgatgcagtcaggggtatgaccaattccacatgagtgtcacctgtacattttattaccactacctccagtttccaaggcagggaagaagaggggaatagtcaaagcaatatgactagctaggtttgggctgctgtttgggctgctgtttttcctggacttttatgccaaactacataaatacttcttggaattcctgatttacgctcaactttgatccctggaccctagggccattagtttccttgaagcagtcctgctggtggatgagcagggaaacttgtagaccctgggaagccaattggtagagggtgcctgcccgccccatcagactgtcccaactggggtgggggtgaggatcatagtgatttctttttaaaagctagtatgactagtgagaatggatgtccaagggcttttctcttcctcccccgagtccacgattcttcatttgtgatcagggttggggtaacttcaccttggtgatcatatattctttttacaaaccaatccagccaagatatatgtgcttttctaaacagcttgtaaaagcaaaaacacaatgtatacacatataaaactgatttttttttttttgcttatatactttgctcaggtggagaacaaggtatgaaagccattatatggtgtcccttggggaccatcccataagtccagggtgttcccaatatacccacaagacaacatgttcaaaacttcatcacctggtccccacaaacctaccaccctcttccattccctcactctggtaaaagcaagccatcttctccagttacgcaaggcagaagcccaggagtgagcacaatgctgccctttcccttgcttctgacttctcatcaatcctattagaactactgactgcaccccacctctcctccatccccacgatcactgccttcatatgaactgcctttcctgtcctctggactttcagtggcttccttttttgctcacctgtccactttcttccccttccctctctccatccctccacatagtcatcagaaagatcatcaaaacaggcaaatgtgactgtattatttccctcacttatctttgtatgcagtttagagtgatttttaagagcacacactttgaaattaggagaacctgtgtctgaatcctgatttttgccatttgtgtgacttgtcaggttatttaatctctttgagcctgtccctcctcagtgaaataggaataataatacctacctcatagagttgttgtgaggattcagtgagatagactgtttctaaagtgtgtagcacagtgcctggcacatagtgggtgctcacattggagcacactattgtcgctatccctgcaggtgtgagttttacatcctttccaagaggcatttgctaataaccctaactaccgcctatccctgtgacactattttctgcctcagcaccctatttcttttgtagacattttctaagtctgtattttatttatttgttggctttttgtctatttctctactagaatgtaagctccatgagagcaaggatgtgtttgtctgatttcccgttacagcccc**TAACGAA^2^**Cgctgagtgactggcacctagtaagcactcagtaactgtttgctgaataaatggaagaatggtcaaaaggctcttcttgatctgatccctgcttgtctgtctggcctcatgctgccactattcctcactgcaccttcagctcaagaaaaacctctttacttgcttctttgaagtgccccttgggcctctgtgtctttgtacctgttgttccctttgcctgaaacaccctttgcctgcccgtctacatggtgaattcctaatcattgatgaagtctcagctcaatggctacctcctctgtgaagctgtccctgatttcttcaggcagatgttctttcccgccttccctgttcccaccgcactttgtaaatacctccatgga**TAACGAA^3^**Ctattgtattgtattgtaatttttttttgcatgtctgtttctactattagactgtaagcaccgtaaaagcagagattgtgctttttcatcctcatgttccccgagcctagcccagtgcctggcacgtaatagcttgtcattaaatatttgttgaatgaatctgtgaatgaacaagagtcatgcagttggaaaagtgttagaagctgtatgtattataaaggaaagaaatgcccttggtggcttccagggtgtttttttttttttttttggtctaaagaggagatctagagagtgttatacaatctctttgaatataagattactcgtttggatttttaaaataatatagatcttcataaataattgcaaagatataaacaagctttgtgatttctcaaatgctatgaagtccaaaataaatatttgcactattagtatttccatagtaaatgctatgagaaaatgtacagcaataaattttgaagctttaaaa |
| **GLIS3 3’ UTR WT** | tccctcttaccctcttcctgactgtggttcatatatccccaccagccgccattgtctgatcatcttgggaaggtagaggtgacagttctcgaaaagtctacagaaagaggcaattctttttctgagttccagtttgaaggcctggaattttcacat**agcactt^1^**ggtcgaaaatatcaagcctaaataggaagtcattttctgtgacacctctacatagctattacagatcctaagtcctagggcatcagctataaaaattggccagtcccaaagacacagtaagttctaacattctggtactatcatatttcaagtattttttctcactttttaaaaatgtttttctgaacaaaagtgaaacaatgccatttatcaaactgagatcattacaatcttcaacaagacaactaaaacagcttgatgttagatgggattctgtgacagtttgtatactgacaggtccttccagtctccctccacagtcccacaagcggaagtattctacctgattccagagttcattccaacatgcagtagaatgaggtgaatgaagaaccaagtgtgggttttgttttcacagtgcacttggtcaatctagtttctctgagtagtgggctgtattctaagaaaagctttcacgtagacgtcaaagtctgcaaaagagatgagttagccactttaaaaaacctgctctgctttacaaaagatgcacaccaggcttcctttacaagtgaactccaaggctgcttgtaaagtaccacccttcctgcagactttgtgggtcgtgtcacttggcctaaacaagtggcctaacatatgcatgctcacagctagtagcatccctcatgcgtcctttgtcatactctggaaagaaaaaaactctgctatcttcagagaaaccatctattcatacttaaatatggaagcctttacaaggaactcaaaatacatttcatttcatttgtacaatgagaaaaaagatgaaaaggcaaggttgttattgcagttgacattgtcagccctgccttttggctttatgtaatttaaaatagaaagggggaaggaggacaagacagaagctcaactttagaaaccatggacatcacctccctctttgagaggagctgcttgtgaaccaggctccagtcttcctgtggcctctcacagtaaccctgcccctttcttacctcacaggggcaagagggactctaaagagggcgtaagcaatagaggcacctgcagccctccttcctcagcaggaaatcaatgtggagccccagagggacattttatctcagagagagcatgctggccacaggacccagtgcagccctgcttcccgcaccgtgggtctttcctgatagctgcaggcttgagattgcagcctcaggataggacacgttcagatgccttccagactacacacactatgcttagattcgagagtctttcctgaccttaaaatttgtcccttgcttactttcacactgcagaagatttatatatatattcatcattatttttgcagcagtacaagtggaaacttcaaaaaaatcattccctttcatgtcctggtctttttaagtcttgatatgtaaccattttcataaatgtccaaacagagattgtcattccccttaaaaacagaaacaaaacacaaaactagtgcttgtgaaaacacacagcgcaaagacaattatcctattgcattaaaaaaaaaatcagtctgcctagagacatttcattggctgcattgtcttaattccctaacgaaactgcatcaaaatgtcagttgtaatttagcctctgatcagctgttaaatattatttaaatatttacaggtcctgtggggtatctcccattacccatgtcgccctcaaattacataagcgatcataaaataagaagaaaaaaaaaggtctttagtagatcacagcagagttaccactgataagagtgttggctttgttcagttgtttctaaattaccagccgtcaagcacagtagcccctaaaaccaattttgagcaaggataaattcaactccctcatctgagaaccttgactttccagttgccttgaagtattgggaatagtaactgtacattttcaatttaattatttggtattttatttgactgtcttggccatgtcattttatggaagcatttttaaat**agcactt^2^**tttgttagtggtgtggaatttctgcggttattatttttttttttttttgcaacagatgaagcaaaaaaaaaaaagcactcattggagaaatagtgagtgtaaaaagagatttattttgtacagacagcaaagtatcctgtgtaatgttgctgacataa**agcactt^3^**tgggggaaaaaagtggaaatctgttttccataaatcacacagactcttgtacatagttatatacactcacgtagagaaatgaactgcatatatcatactggatatggggccgattttactctagaggcacatctggtgcttattgtcata |
| **GLIS3 3’ UTR Mut** | tccctcttaccctcttcctgactgtggttcatatatccccaccagccgccattgtctgatcatcttgggaaggtagaggtgacagttctcgaaaagtctacagaaagaggcaattctttttctgagttccagtttgaaggcctggaattttcacat**taacgaa^1^**ggtcgaaaatatcaagcctaaataggaagtcattttctgtgacacctctacatagctattacagatcctaagtcctagggcatcagctataaaaattggccagtcccaaagacacagtaagttctaacattctggtactatcatatttcaagtattttttctcactttttaaaaatgtttttctgaacaaaagtgaaacaatgccatttatcaaactgagatcattacaatcttcaacaagacaactaaaacagcttgatgttagatgggattctgtgacagtttgtatactgacaggtccttccagtctccctccacagtcccacaagcggaagtattctacctgattccagagttcattccaacatgcagtagaatgaggtgaatgaagaaccaagtgtgggttttgttttcacagtgcacttggtcaatctagtttctctgagtagtgggctgtattctaagaaaagctttcacgtagacgtcaaagtctgcaaaagagatgagttagccactttaaaaaacctgctctgctttacaaaagatgcacaccaggcttcctttacaagtgaactccaaggctgcttgtaaagtaccacccttcctgcagactttgtgggtcgtgtcacttggcctaaacaagtggcctaacatatgcatgctcacagctagtagcatccctcatgcgtcctttgtcatactctggaaagaaaaaaactctgctatcttcagagaaaccatctattcatacttaaatatggaagcctttacaaggaactcaaaatacatttcatttcatttgtacaatgagaaaaaagatgaaaaggcaaggttgttattgcagttgacattgtcagccctgccttttggctttatgtaatttaaaatagaaagggggaaggaggacaagacagaagctcaactttagaaaccatggacatcacctccctctttgagaggagctgcttgtgaaccaggctccagtcttcctgtggcctctcacagtaaccctgcccctttcttacctcacaggggcaagagggactctaaagagggcgtaagcaatagaggcacctgcagccctccttcctcagcaggaaatcaatgtggagccccagagggacattttatctcagagagagcatgctggccacaggacccagtgcagccctgcttcccgcaccgtgggtctttcctgatagctgcaggcttgagattgcagcctcaggataggacacgttcagatgccttccagactacacacactatgcttagattcgagagtctttcctgaccttaaaatttgtcccttgcttactttcacactgcagaagatttatatatatattcatcattatttttgcagcagtacaagtggaaacttcaaaaaaatcattccctttcatgtcctggtctttttaagtcttgatatgtaaccattttcataaatgtccaaacagagattgtcattccccttaaaaacagaaacaaaacacaaaactagtgcttgtgaaaacacacagcgcaaagacaattatcctattgcattaaaaaaaaaatcagtctgcctagagacatttcattggctgcattgtcttaattccctaacgaaactgcatcaaaatgtcagttgtaatttagcctctgatcagctgttaaatattatttaaatatttacaggtcctgtggggtatctcccattacccatgtcgccctcaaattacataagcgatcataaaataagaagaaaaaaaaaggtctttagtagatcacagcagagttaccactgataagagtgttggctttgttcagttgtttctaaattaccagccgtcaagcacagtagcccctaaaaccaattttgagcaaggataaattcaactccctcatctgagaaccttgactttccagttgccttgaagtattgggaatagtaactgtacattttcaatttaattatttggtattttatttgactgtcttggccatgtcattttatggaagcatttttaaat**taacgaa^2^**tttgttagtggtgtggaatttctgcggttattatttttttttttttttgcaacagatgaagcaaaaaaaaaaaagcactcattggagaaatagtgagtgtaaaaagagatttattttgtacagacagcaaagtatcctgtgtaatgttgctgacataa**taacgaa^3^**tgggggaaaaaagtggaaatctgttttccataaatcacacagactcttgtacatagttatatacactcacgtagagaaatgaactgcatatatcatactggatatggggccgattttactctagaggcacatctggtgcttattgtcata |

The 3 putitive binding sties of MBD2 (NM_333927.5) located at 1: 1641-1648，2: 4179-4186 and 3: 4566-4572. The 3 putitive binding sties of GLIS3 (NM_001042413.2) located at 1: 4814-4870，2: 6836-6842 and 3: 7001-7007.

**Supplementary Table S6. Primers used for** **quantitative PCR (qPCR).**

| **Name** | **Sequence** |  |
| --- | --- | --- |
| For quantification of gene expression | | |
| GAPDH | Forward (5’-3’) | GGAGCGAGATCCCTCCAAAAT |
|  | Reverse (5’-3’) | GGCTGTTGTCATACTTCTCATGG |
| U6 | Forward (5’-3’) | GGAACGATACAGAGAAGATTAGC |
|  | Reverse (5’-3’) | TGGAACGCTTCACGAATTTGCG |
| circKCNN2 | Forward (5’-3’) | CTGTCCGAGCTTGTGAAAGT |
|  | Reverse (5’-3’) | TGGTTGTGGATGGGGCATAG |
| MBD2 | Forward (5’-3’) | ATACAGAAGAGATGGATATTGAA |
|  | Reverse (5’-3’) | GGCATTGGTTAGTGCTATT |
| JUNB | Forward (5’-3’) | CCAAGAGCGCATCAAAGTGG |
|  | Reverse (5’-3’) | GTTCAGAAGGCGTGTCCCTT |
| NFYA | Forward (5’-3’) | AGTCCAGACCCTCCAGGTAGTC |
|  | Reverse (5’-3’) | CCATCATGACCATCCCTCCTG |
| KCNN2 | Forward (5’-3’) | ACACTTTGGTGGACTTGGCA |
|  | Reverse (5’-3’) | AATCTCTCTGCTGCTGCCTG |
| For ChIP followed qPCR or RNA pull-down followed qPCR | | |
| KCNN2 | Forward (5’-3’) | TGCAGCTTTAACAGCCCTGA |
|  | Reverse (5’-3’) | GCAGGGACAGGTTATCTCCC |
| MBD2 binding site 1 | Forward (5’-3’) | GCTTTTGATGCCAGGTGCAA |
|  | Reverse (5’-3’) | AAACTGTGGGGGAAACAAATG |
| MBD2 binding site 2 | Forward (5’-3’) | CTATTTTCTGCCTCAGCACCC |
|  | Reverse (5’-3’) | TGAGCTGAAGGTGCAGTGAG |
| MBD2 binding site 3 | Forward (5’-3’) | GCTTCTTTGAAGTGCCCCTTG |
|  | Reverse (5’-3’) | CACCAAGGGCATTTCTTTCCT |

**Supplementary Table S7. Gene sets enriched by circKCNN2 in patients with HCC (n=13).**

| **Gene set** | **Size** | **ES** | **NES** | **p value** | **FDR** |
| --- | --- | --- | --- | --- | --- |
| FLORIO_NEOCORTEX_BASAL_RADIAL_GLIA_DN | 150 | -0.64 | -2.09 | 0.02 | 0.23 |
| GRAHAM_CML_DIVIDING_VS_NORMAL_QUIESCENT_UP | 148 | -0.58 | -2.03 | 0.02 | 0.20 |
| BOHN_PRIMARY_IMMUNODEFICIENCY_SYNDROM_UP | 42 | -0.54 | -2.03 | 0.00 | 0.16 |
| LE_EGR2_TARGETS_UP | 94 | -0.64 | -2.02 | 0.01 | 0.15 |
| GRAHAM_NORMAL_QUIESCENT_VS_NORMAL_DIVIDING_DN | 72 | -0.72 | -2.00 | 0.01 | 0.17 |
| MORI_IMMATURE_B_LYMPHOCYTE_DN | 75 | -0.69 | -1.99 | 0.01 | 0.17 |
| LEE_EARLY_T_LYMPHOCYTE_UP | 82 | -0.65 | -1.99 | 0.01 | 0.14 |
| BURTON_ADIPOGENESIS_PEAK_AT_24HR | 39 | -0.71 | -1.98 | 0.01 | 0.14 |
| SHEPARD_BMYB_TARGETS | 53 | -0.60 | -1.98 | 0.01 | 0.13 |
| NAKAYAMA_SOFT_TISSUE_TUMORS_PCA2_UP | 53 | -0.67 | -1.95 | 0.04 | 0.16 |
| RHODES_UNDIFFERENTIATED_CANCER | 59 | -0.71 | -1.95 | 0.01 | 0.15 |
| CROONQUIST_IL6_DEPRIVATION_DN | 85 | -0.72 | -1.95 | 0.01 | 0.15 |
| BENPORATH_PROLIFERATION | 131 | -0.67 | -1.92 | 0.01 | 0.20 |
| MORI_LARGE_PRE_BII_LYMPHOCYTE_UP | 75 | -0.68 | -1.91 | 0.01 | 0.20 |
| PETROVA_PROX1_TARGETS_UP | 23 | -0.67 | -1.91 | 0.00 | 0.20 |
| MORI_PRE_BI_LYMPHOCYTE_UP | 68 | -0.56 | -1.90 | 0.02 | 0.20 |
| BURTON_ADIPOGENESIS_3 | 82 | -0.69 | -1.90 | 0.02 | 0.19 |
| ZHOU_CELL_CYCLE_GENES_IN_IR_RESPONSE_6HR | 68 | -0.70 | -1.90 | 0.02 | 0.18 |
| BOYAULT_LIVER_CANCER_SUBCLASS_G123_UP | 45 | -0.79 | -1.89 | 0.00 | 0.18 |
| ROSTY_CERVICAL_CANCER_PROLIFERATION_CLUSTER | 114 | -0.73 | -1.89 | 0.03 | 0.18 |
| SOTIRIOU_BREAST_CANCER_GRADE_1_VS_3_UP | 126 | -0.74 | -1.89 | 0.01 | 0.18 |
| MORI_MATURE_B_LYMPHOCYTE_DN | 65 | -0.52 | -1.89 | 0.00 | 0.17 |
| KANG_DOXORUBICIN_RESISTANCE_UP | 47 | -0.81 | -1.88 | 0.01 | 0.18 |
| WHITFIELD_CELL_CYCLE_LITERATURE | 39 | -0.75 | -1.88 | 0.03 | 0.17 |
| WU_APOPTOSIS_BY_CDKN1A_VIA_TP53 | 42 | -0.71 | -1.88 | 0.01 | 0.17 |
| NADERI_BREAST_CANCER_PROGNOSIS_UP | 31 | -0.63 | -1.87 | 0.02 | 0.17 |
| ZHOU_CELL_CYCLE_GENES_IN_IR_RESPONSE_24HR | 105 | -0.65 | -1.87 | 0.02 | 0.17 |
| NAKAMURA_CANCER_MICROENVIRONMENT_DN | 37 | -0.60 | -1.85 | 0.01 | 0.19 |
| KOBAYASHI_EGFR_SIGNALING_24HR_DN | 208 | -0.61 | -1.85 | 0.03 | 0.19 |
| WEST_ADRENOCORTICAL_TUMOR_UP | 274 | -0.54 | -1.85 | 0.00 | 0.19 |
| SIMBULAN_PARP1_TARGETS_DN | 16 | -0.78 | -1.85 | 0.00 | 0.19 |
| ZHAN_MULTIPLE_MYELOMA_SUBGROUPS | 30 | -0.65 | -1.83 | 0.00 | 0.21 |
| GAVIN_FOXP3_TARGETS_CLUSTER_P6 | 81 | -0.53 | -1.83 | 0.03 | 0.21 |
| DUTERTRE_ESTRADIOL_RESPONSE_24HR_UP | 269 | -0.55 | -1.82 | 0.05 | 0.21 |
| CROONQUIST_NRAS_SIGNALING_DN | 63 | -0.72 | -1.82 | 0.03 | 0.21 |
| MANALO_HYPOXIA_DN | 271 | -0.53 | -1.82 | 0.00 | 0.21 |
| MATTIOLI_MGUS_VS_PCL | 114 | -0.49 | -1.82 | 0.01 | 0.20 |
| SARRIO_EPITHELIAL_MESENCHYMAL_TRANSITION_UP | 153 | -0.57 | -1.81 | 0.04 | 0.22 |
| VECCHI_GASTRIC_CANCER_EARLY_UP | 357 | -0.49 | -1.80 | 0.01 | 0.23 |
| ODONNELL_TFRC_TARGETS_DN | 101 | -0.58 | -1.80 | 0.03 | 0.24 |
| SUNG_METASTASIS_STROMA_DN | 49 | -0.52 | -1.80 | 0.01 | 0.24 |
| ODONNELL_TARGETS_OF_MYC_AND_TFRC_DN | 39 | -0.70 | -1.80 | 0.02 | 0.24 |
| BOYAULT_LIVER_CANCER_SUBCLASS_G3_UP | 184 | -0.61 | -1.79 | 0.00 | 0.24 |
| ISHIDA_E2F_TARGETS | 44 | -0.74 | -1.79 | 0.04 | 0.23 |
| BERENJENO_TRANSFORMED_BY_RHOA_UP | 493 | -0.47 | -1.79 | 0.01 | 0.23 |
| EGUCHI_CELL_CYCLE_RB1_TARGETS | 20 | -0.91 | -1.78 | 0.01 | 0.24 |
| LUI_THYROID_CANCER_PAX8_PPARG_DN | 44 | -0.51 | -1.78 | 0.01 | 0.24 |
| BURTON_ADIPOGENESIS_PEAK_AT_16HR | 37 | -0.60 | -1.78 | 0.02 | 0.23 |
| WANG_RESPONSE_TO_GSK3_INHIBITOR_SB216763_DN | 310 | -0.44 | -1.78 | 0.00 | 0.23 |
| SONG_TARGETS_OF_IE86_CMV_PROTEIN | 50 | -0.67 | -1.78 | 0.04 | 0.22 |
| CHEMNITZ_RESPONSE_TO_PROSTAGLANDIN_E2_UP | 131 | -0.55 | -1.78 | 0.02 | 0.23 |
| REN_BOUND_BY_E2F | 50 | -0.70 | -1.78 | 0.03 | 0.23 |
| WHITEFORD_PEDIATRIC_CANCER_MARKERS | 90 | -0.64 | -1.78 | 0.05 | 0.23 |
| SCIAN_CELL_CYCLE_TARGETS_OF_TP53_AND_TP73_DN | 21 | -0.77 | -1.77 | 0.03 | 0.22 |
| ZHAN_MULTIPLE_MYELOMA_PR_UP | 37 | -0.82 | -1.77 | 0.02 | 0.22 |
| KONG_E2F3_TARGETS | 76 | -0.68 | -1.77 | 0.06 | 0.22 |
| SHEDDEN_LUNG_CANCER_POOR_SURVIVAL_A6 | 379 | -0.54 | -1.77 | 0.01 | 0.23 |
| FOURNIER_ACINAR_DEVELOPMENT_LATE_2 | 261 | -0.52 | -1.77 | 0.01 | 0.22 |
| WEST_ADRENOCORTICAL_TUMOR_MARKERS_UP | 20 | -0.71 | -1.76 | 0.03 | 0.23 |
| RICKMAN_TUMOR_DIFFERENTIATED_MODERATELY_VS_POORLY_DN | 15 | -0.71 | -1.76 | 0.00 | 0.23 |
| GRADE_COLON_AND_RECTAL_CANCER_UP | 266 | -0.48 | -1.75 | 0.00 | 0.24 |
| RHEIN_ALL_GLUCOCORTICOID_THERAPY_DN | 341 | -0.44 | -1.75 | 0.02 | 0.25 |
| FERREIRA_EWINGS_SARCOMA_UNSTABLE_VS_STABLE_UP | 144 | -0.53 | -1.75 | 0.02 | 0.24 |

Abbreviation: ES, enrichment score; NES, normalized enrichment score; FDR, false discovery rate.

**Supplementary Table S8. The potential target miRNAs predicted by RegRNA2.0 of circKCNN2.**

| **miRNA Name** | **Position** | **Length** | **Score** | **Sequence** |
| --- | --- | --- | --- | --- |
| hsa-miR-520b | 268~292 | 25 | 173 | TCCTCTAGAAGCATTGGAGCACTTA |
| hsa-miR-302c-3p | 269~292 | 24 | 176 | CCTCTAGAAGCATTGGAGCACTTA |
| hsa-miR-3945 | 136~158 | 23 | 177 | CGGCTTGCCTTCTCCTATGCCCC |
| hsa-miR-520c-3p | 267~292 | 26 | 178 | CTCCTCTAGAAGCATTGGAGCACTTA |

**Supplementary Table S9. The clinical information of HCC patients that organoids derived from.**

| **HCC organoids ID** | **Org543** | **Org801** |
| --- | --- | --- |
| Pathology Diagnosis | HCC | HCC |
| Age | 49 | 48 |
| Gender | female | male |
| HbsAg | positive | positive |
| Child-pugh stage | A | A |
| Tumor Number | 1 | 1 |
| Total Tumor size (cm) | 13 | 10.6 |
| HCC Edmondson | 3 | 3 |
| MVI | 2 | 1 |
| TNM stage | II | II |
| BCLC stage | C | C |
| Recurrence | No | Yes |

MVI: microvascular invasion; BCLC: Barcelona Clinic Liver Cancer.
