# Supplementary figures and images for: circKCNN2 suppresses the recurrence of hepatocellular carcinoma at least partially via regulating miR‐520c‐3p/methyl‐DNA‐binding domain protein 2 axis
Source: Clin Transl Med. 2022 Jan 20;12(1):e662. doi: 10.1002/ctm2.662 (PMC8775140; doi:10.1002/ctm2.662)

A

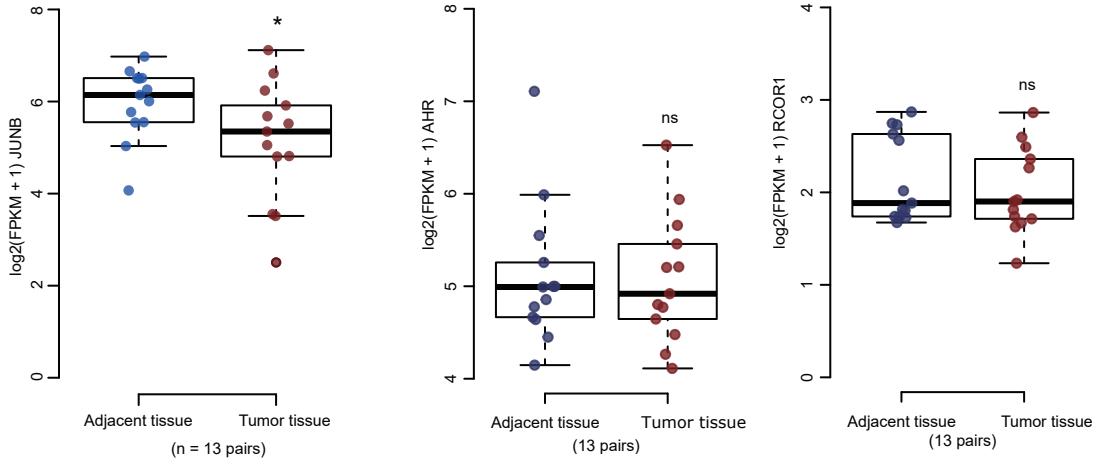

B

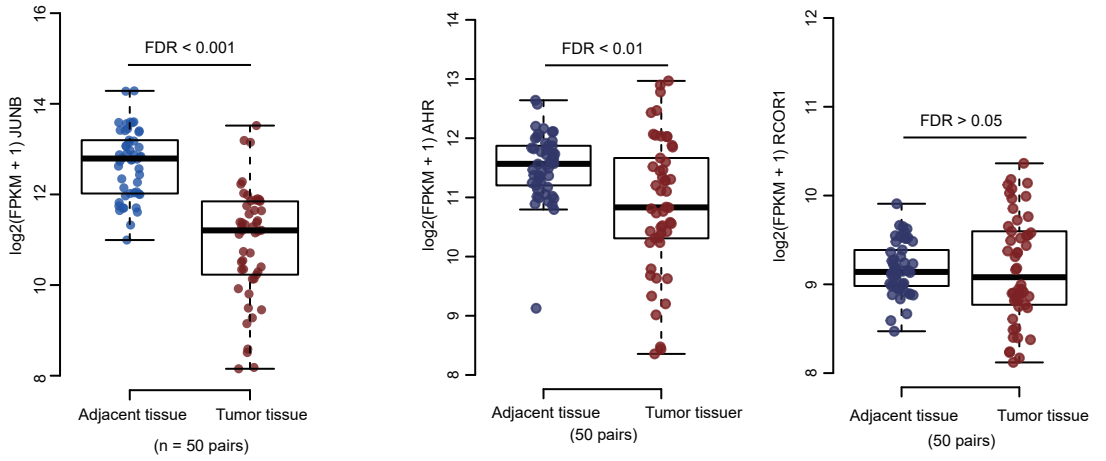

C

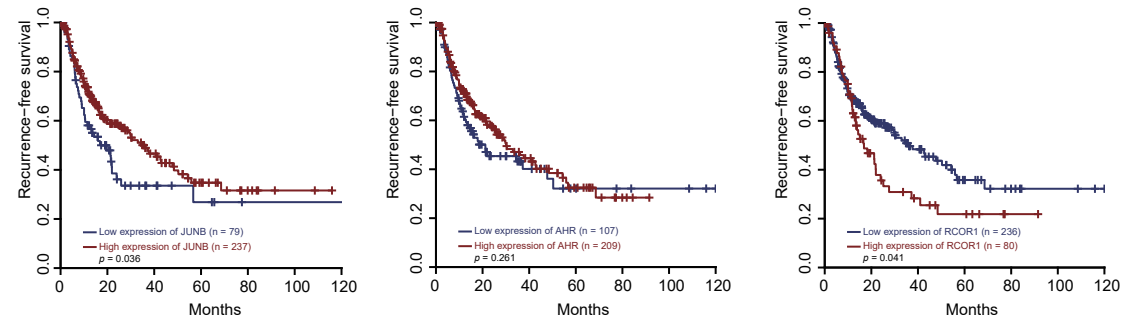

D

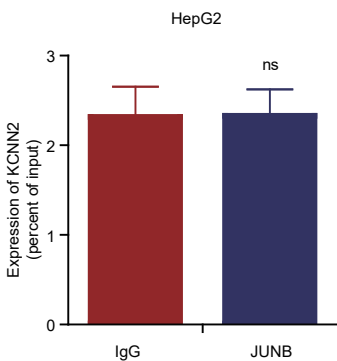

E

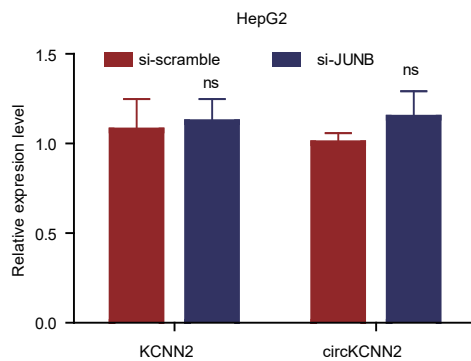

Supplement: Supplementary file 1 — Supporting information [file CTM2-12-e662-s001.zip › Supplementary Figure S1.pdf]

# Supplementary Figure S2

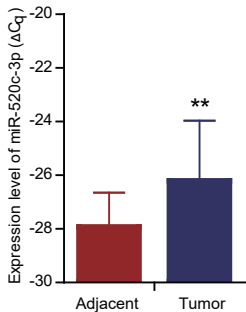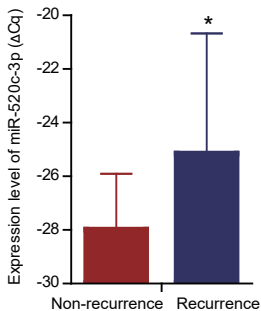

Supplement: Supplementary file 1 — Supporting information [file CTM2-12-e662-s001.zip › Supplementary Figure S2.pdf]

# Supplementary Figure S3

A

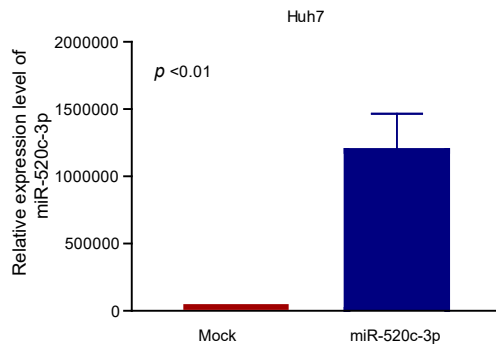

B

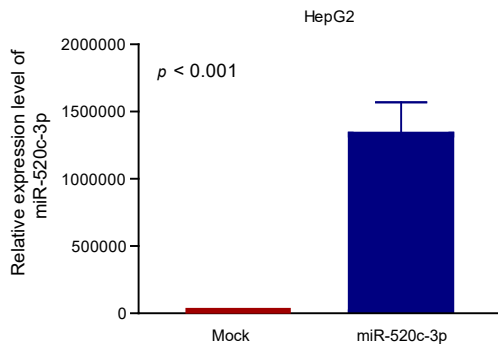

C

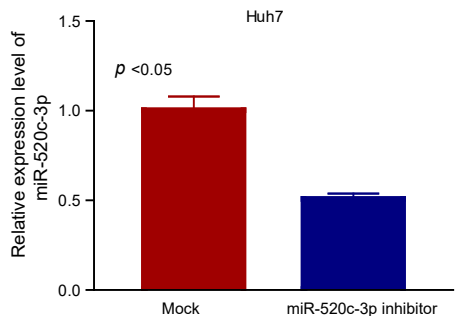

D

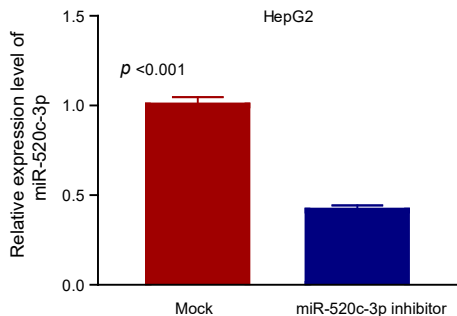

Supplement: Supplementary file 1 — Supporting information [file CTM2-12-e662-s001.zip › Supplementary Figure S3.pdf]

# Supplementary Figure S4

A

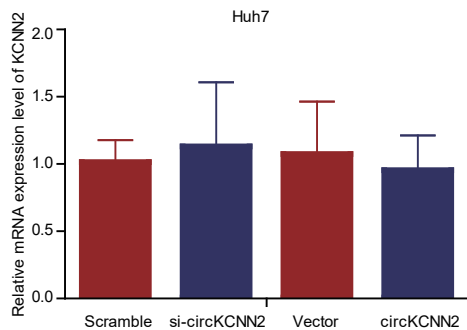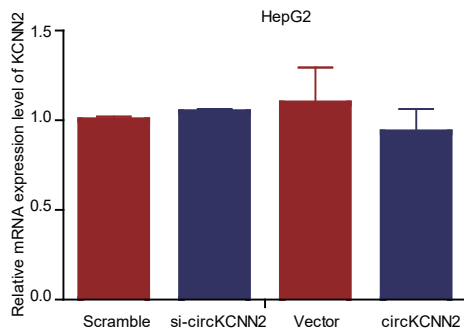

B

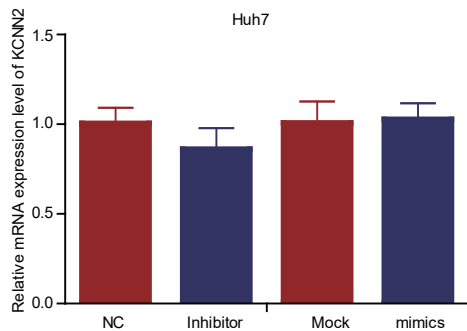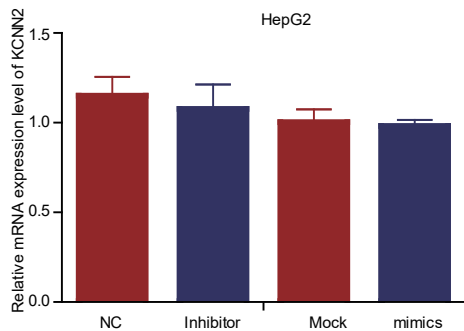

Supplement: Supplementary file 1 — Supporting information [file CTM2-12-e662-s001.zip › Supplementary Figure S4.pdf]

# Supplementary Figure S5

A

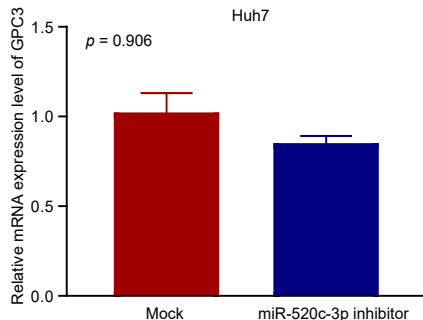

B

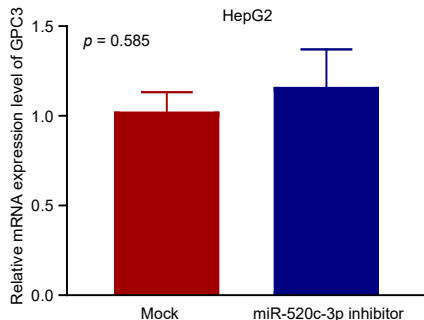

C

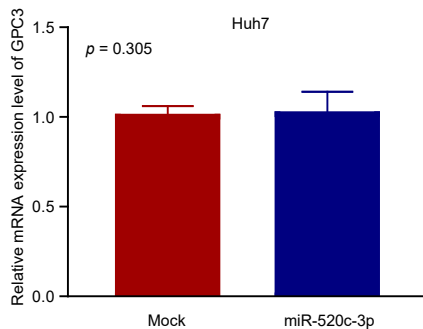

D

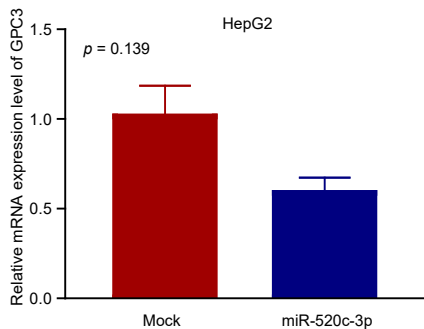

Supplement: Supplementary file 1 — Supporting information [file CTM2-12-e662-s001.zip › Supplementary Figure S5.pdf]

# Supplementary Figure S7

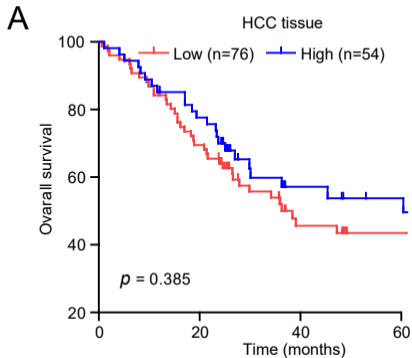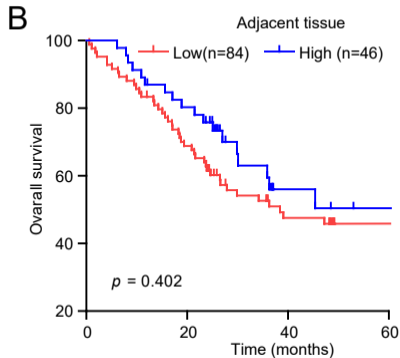

Supplement: Supplementary file 1 — Supporting information [file CTM2-12-e662-s001.zip › Supplementary Figure S7.pdf]

A

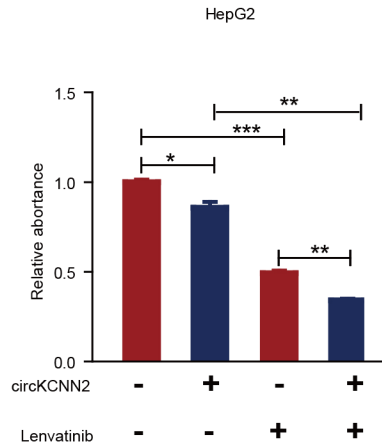

B

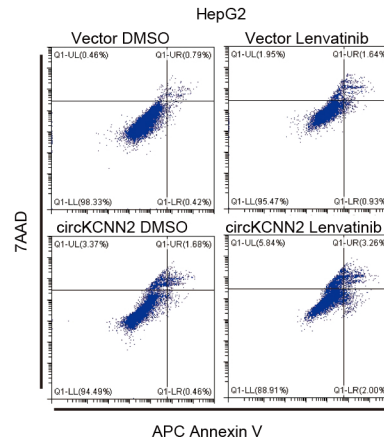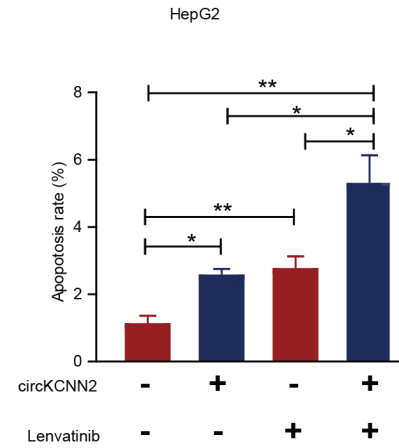

C

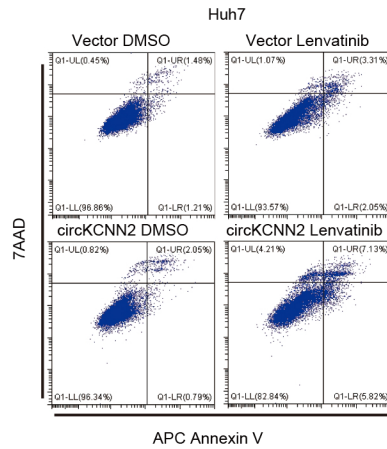

D

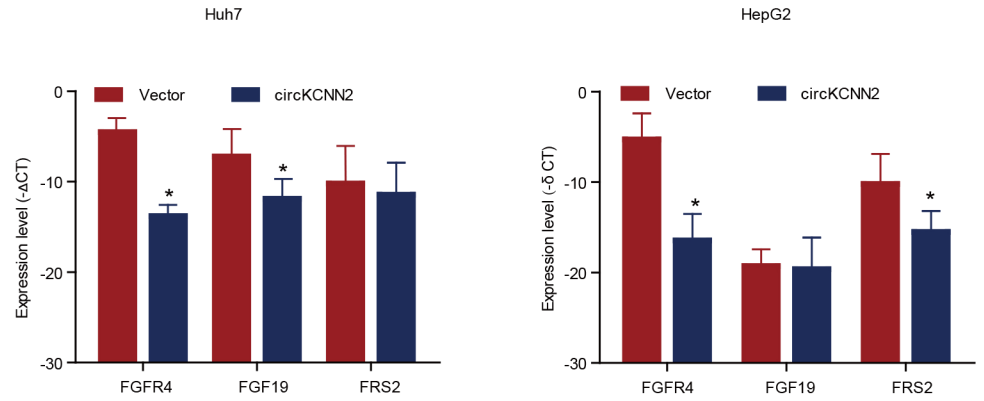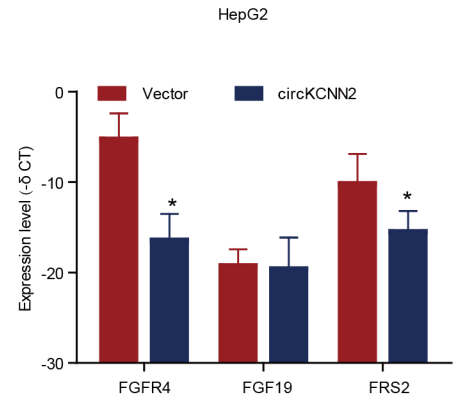

E

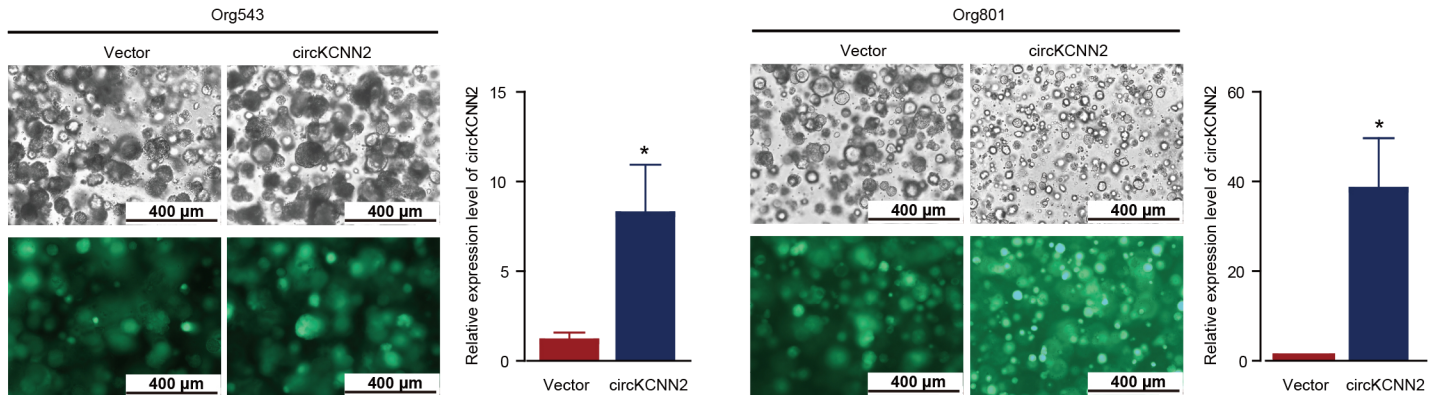

Supplement: Supplementary file 1 — Supporting information [file CTM2-12-e662-s001.zip › Supplementary Figure S8.pdf]
